# Gut Dysbiosis, Uremic Toxins, and NLRP3 Signaling in Chronic Kidney Disease: From Mechanistic Evidence to Clinical Biomarker Evaluation

**DOI:** 10.3390/diagnostics16193084

**Published:** 2026-09-23

**Authors:** Imola Donath-Miklos, Oana Știrbu, Romana Olivia Popețiu, Adrian Silviu Crișan, Paula Alexandra Vulciu, Roxana Andra Coman, Cecilia Roberta Avram, Darius Radu Roman, Radmila-Anca Bugari, Dana Zdremțan, Maria Orodan, Luminița Pilat, Larisa Alexandra Rus, Cristina Georgiana Firu, Mihai Cătălin Roșu, Simona Maria Borta

**Affiliations:** 1 Department of Biology and Life Science, Faculty of Medicine, “Vasile Goldiș” Western University of Arad, B-dul Revoluției nr. 94-96, 310025 Arad, Romania; miklos.imola@uvvg.ro (I.D.-M.); avramcici02@yahoo.com (C.R.A.); orodan.maria@uvvg.ro (M.O.); 2 Department of Internal Medicine, Faculty of Medicine, “Vasile Goldiș” Western University of Arad, B-dul Revoluției nr. 94-96, 310025 Arad, Romania; oanastirbu66@yahoo.com (O.Ș.); larisa_gal@yahoo.com (L.A.R.); simoborta@yahoo.com (S.M.B.); 3 Hemodialysis Department, Arad County Emergency Clinical Hospital, Str. Andrényi Károly nr 2-4, 310037 Arad, Romania; 4 Internal Medicine Clinic, Arad County Emergency Clinical Hospital, Str. Andrényi Károly nr 2-4, 310037 Arad, Romania; 5 Department of Intensive Care and Emergency Medicine, Faculty of Medicine, “Vasile Goldiș” Western University of Arad, B-dul Revoluției nr. 94-96, 310025 Arad, Romania; 6 Intensive Care and Emergency Medicine Clinic, Arad County Emergency Clinical Hospital, Str. Andrényi Károly nr 2-4, 310037 Arad, Romania; 7 Department of Biochemistry, Faculty of Medicine, “Vasile Goldiș” Western University of Arad, B-dul Revoluției nr. 94-96, 310025 Arad, Romania; vulciu.paula@uvvg.ro (P.A.V.); zdremtan.dana@uvvg.ro (D.Z.); luminita.pilat@yahoo.com (L.P.); 8 Department of Urology, MedLife Humanitas Hospital, 400664 Cluj-Napoca, Romania; dr.roxanacoman@yahoo.com; 9 Fundamental Discipline, Anatomy, Faculty of Nursing and Health Sciences, “Iuliu Hațieganu” University of Medicine and Pharmacy, Victor Babes Street Number 8, 400347 Cluj-Napoca, Romania; 10 Clinical Laboratory of Medical Analyses, Arad County Emergency Clinical Hospital, Str. Andrényi Károly nr 2-4, 310037 Arad, Romania; 11 Doctoral School of Biomedical Sciences, Faculty of Medicine and Pharmacy, University of Oradea, Str. Universității nr 1, 410087 Oradea, Romania; roman.dariusradu@student.uoradea.ro; 12 Department of Otorhinolaringology, Faculty of Medicine, “Vasile Goldiș” Western University of Arad, B-dul Revoluției nr. 94-96, 310025 Arad, Romania; radmilabugariturcin@gmail.com; 13 Department of Heamatology, Faculty of Medicine, “Vasile Goldiș” Western University of Arad, B-dul Revoluției nr. 94-96, 310025 Arad, Romania; firu.cristina@uvvg.ro; 14 Department of General Surgery, Faculty of Medicine, “Vasile Goldiș” Western University of Arad, B-dul Revoluției nr. 94-96, 310025 Arad, Romania; rosu.mihai@uvvg.ro

**Keywords:** gut-kidney axis, gut-derived uremic toxins, LPS, IS, p-CS, NLRP-3 inflammasome, TMAO

## Abstract

Chronic kidney disease (CKD) is associated with intestinal dysbiosis, barrier dysfunction, uremic toxin accumulation, and systemic inflammation. This narrative review critically evaluates the gut–kidney axis, focusing on the distinction between NLRP3 [Nucleotide-binding Oligomerization Domain (NOD)-like receptor pyrin domain-containing protein 3] inflammasome priming and direct activation. It also examines the diagnostic and prognostic relevance of related biomarkers. Preclinical studies, clinical investigations, systematic reviews, meta-analyses, and relevant clinical trials within the past decade were considered. CKD-associated barrier dysfunction increases exposure to lipopolysaccharides and gut-derived metabolites. Lipopolysaccharide (LPS)-mediated TLR4/NF-κB (Toll-like receptor 4/nuclear factor kappa B) signaling provides well-supported inflammasome priming. Indoxyl sulfate acts predominantly as a priming stimulus, although its effects vary among cell types. Direct NLRP3 activation by p-cresyl sulfate has not been demonstrated. Trimethylamine N-oxide has the strongest experimental evidence for inflammasome activation. Candidate biomarkers include these metabolites, microbial profiles, intestinal permeability markers, and inflammasome-related mediators. However, their interpretation is limited by dependence on renal clearance, assay heterogeneity, absent standardized thresholds, and uncertain added value beyond estimated glomerular filtration rate and urinary albumin-to-creatinine ratio. Interventional studies remain small and predominantly use surrogate endpoints. The gut–toxin–inflammasome axis is mechanistically plausible, but evidence for priming is substantially stronger than that for direct activation. Its biomarkers remain research tools and require assay harmonization, prospective validation, and demonstration of independent clinical value before routine implementation.

## 1. Introduction

Chronic kidney disease (CKD) affects approximately 10–14% of the global population, and its prevalence continues to rise in parallel with population aging, diabetes mellitus, hypertension, obesity, and metabolic syndrome. Beyond progressive loss of renal function, CKD is strongly associated with cardiovascular complications, chronic inflammation, endothelial dysfunction, and increased all-cause mortality [1]. In advanced CKD, particularly among older patients, death represents an important competing outcome that may precede progression to kidney failure, emphasizing the importance of early risk reduction [2,3]. Current CKD treatment targets the modifiable underlying cause, slows the final common pathway of nephron loss, and manages systemic and downstream risk. Gut dysbiosis—a potential “midstream” driver of progression through proinflammatory and pro-fibrotic mechanisms—has not yet been validated as a therapeutic target and remains absent from standard treatment guidelines. A clinically established gut-targeted example is targeted-release budesonide for IgA nephropathy, which acts on ileal gut-associated lymphoid tissue and is included in current KDIGO (Kidney Disease: Improving Global Outcomes) guidance [4,5,6,7,8,9]. Recently, the gut-kidney axis has undergone a conceptual shift from a phenomenon viewed merely as a consequence of chronic kidney disease to a recognized contributor to toxin generation, immune dysregulation and extrarenal complications of CKD [10].

Chronic low-grade inflammation is central to this process. Persistent inflammatory signaling contributes to endothelial dysfunction, podocyte injury, tubular damage, oxidative stress, and extracellular matrix accumulation, ultimately promoting glomerulosclerosis and tubulointerstitial fibrosis [11,12]. Among the pathways implicated, nuclear factor kappa B (NF-κB) signaling and activation of the NOD-like receptor pyrin domain-containing protein 3 (NLRP3) inflammasome have emerged as key mediators. NLRP3 signaling in CKD is initiated by priming signals such as bacterial endotoxins and activated by danger signals including extracellular ATP (adenosine triphosphate), urate crystals, mitochondrial dysfunction, and reactive oxygen species (ROS). Once activated, the NLRP3 inflammasome drives caspase-1-mediated maturation of interleukin (IL)-1β and IL-18, amplifying renal injury and promoting pyroptotic cell death. Increased expression of NLRP3-related components has been demonstrated in renal tubular epithelial cells, podocytes, mesangial cells, and infiltrating macrophages during CKD progression [11,12,13,14].

In parallel, the gut–kidney axis has emerged as an important pathophysiological framework linking intestinal homeostasis to renal function and systemic inflammation. The intestinal microbiota contributes to nutrient metabolism, immune regulation, epithelial barrier integrity, and production of bioactive metabolites, including short-chain fatty acids (SCFAs). Alterations in microbial composition and diversity, termed dysbiosis, disrupt barrier function and promote systemic dissemination of bacterial endotoxins and uremic toxins; CKD itself alters microbial composition, establishing a bidirectional relationship between renal dysfunction and intestinal dysbiosis [15]. Patients with CKD show increased intestinal permeability with disruption of tight junction proteins, including claudins, occludin, and zonula occludens-1 (ZO-1), facilitating translocation of lipopolysaccharides (LPSs) and microbe-associated molecular patterns into the systemic circulation, while a dysbiotic microbiota increases production of protein-bound uremic toxins such as indoxyl sulfate (IS) and p-cresyl sulfate (pCS) [10,16,17].

These two lines of evidence converge. Gut-derived endotoxemia and dysbiosis-associated metabolites may contribute to NLRP3 signaling, although their roles differ between inflammasome priming and direct activation. LPSsTMAO activate Toll-like receptor 4 (TLR4) and NF-κB-dependent priming. IS and pCS promote oxidative stress and NF-κB-dependent inflammatory priming; however, evidence that either metabolite directly provides the second NLRP3 activation signal remains limited and inconsistent [12,18,19,20,21]. This junction between microbial metabolism and innate immune activation remains the least well characterized element of the gut–kidney axis, and it is the specific focus of the present review.

Altered gut microbial profiles have been described across several CKD etiologies—including IgA nephropathy, diabetic kidney disease, membranous nephropathy, and nephrolithiasis—connected to renal health through metabolic, immune and inflammatory pathways, which may contribute to kidney injury before CKD is established [17,22,23]. However, causal and temporal relationships remain uncertain, and dysbiosis has not been established as an independent driver of any specific kidney condition [23].

This narrative review critically evaluates the role of gut dysbiosis, intestinal barrier dysfunction, uremic toxins, and NLRP3 inflammasome activation in CKD pathophysiology, focusing specifically on whether components of the gut–toxin–NLRP3 axis have progressed from mechanistic observations to clinically useful diagnostic or prognostic biomarkers. We distinguish inflammasome priming from direct activation, assess the human evidence for IS, pCS, TMAO, and downstream inflammatory mediators, and examine the analytical, pre-analytical, and clinical-validation barriers to their use alongside established measures such as eGFR (estimated glomerular filtration rate) and albuminuria. Therapeutic modulation of this axis as a tool for biomarker-guided patient stratification or treatment monitoring is also discussed, alongside current translational challenges in microbiome-based interventions.

## 2. Materials and Methods

### 2.1. Search Strategy

A narrative literature search was conducted in PubMed/MEDLINE, Scopus, and Web of Science between 1 June 2026 and 12 September 2026, with the final search performed on 12 September 2026. English-language articles published between January 2000 and September 2026 were considered. Representative search combinations included (“chronic kidney disease” OR CKD) AND (“gut microbiota” OR “gut microbiome” OR dysbiosis OR “intestinal permeability” OR “uremic toxins” OR “indoxyl sulfate” OR “p-cresyl sulfate” OR “trimethylamine N-oxide” OR TMAO OR NLRP3 OR inflammasome OR pyroptosis OR “renal fibrosis”). The search was applied to titles and abstracts in PubMed/MEDLINE, to TITLE-ABS-KEY in Scopus, and as a topic search (TS) in Web of Science.

Original experimental and clinical studies, systematic reviews, meta-analyses, and relevant narrative reviews addressing the gut–kidney axis, uremic toxins, NLRP3 signaling, associated biomarkers, or related interventions in CKD were considered eligible. Non-English publications, duplicate records, articles published before 2000, and studies without clear relevance to CKD or the investigated pathways were excluded. Reference lists of relevant articles were screened manually. ClinicalTrials.gov was searched for ongoing and completed studies involving microbiome-directed interventions, uremic toxin reduction, or NLRP3 inhibition in CKD. Trial registrations without published results were retained only to describe ongoing research. As this was a narrative review, no formal risk-of-bias assessment or meta-analysis was performed.

Given the narrative nature of this review, evidence was interpreted qualitatively according to study design. In vitro and animal studies were used to support mechanistic plausibility, observational human studies to describe clinical associations, and randomized trials and meta-analyses to evaluate therapeutic efficacy. Trial registrations without published results were considered evidence of ongoing research rather than clinical efficacy.

### 2.2. Generative AI Use

During manuscript preparation, the authors used ChatGPT (OpenAI, GPT-5.6 Sol, San Francisco, CA, USA) for English-language editing, structural reorganization, and the preliminary development of schematic figure concepts. All AI-assisted content was reviewed and edited by the authors. Scientific statements and references were checked against the original sources. Generative AI was not used to generate or analyze clinical or experimental data. The authors take full responsibility for the content of the manuscript.

## 3. Results

### 3.1. Physiology of the Gut–Kidney Axis

#### 3.1.1. Intestinal Microbiota in Healthy Individuals

The healthy gastrointestinal tract contains a diverse community of bacteria, archaea, viruses, and fungi. These microorganisms support digestion, vitamin synthesis, immune maturation, and epithelial barrier integrity. *Firmicutes* and *Bacteroidetes* are commonly among the most abundant bacterial phyla, while *Actinobacteria*, *Proteobacteria*, and *Verrucomicrobia* are generally represented at lower relative abundances. Microbiota composition varies with age, diet, genetics, medication use, environmental exposure, and lifestyle. This variability must be considered when microbiome profiles are evaluated as potential clinical biomarkers [24,25].

Commensal microorganisms interact with intestinal epithelial cells, dendritic cells, macrophages, and gut-associated lymphoid tissue. These interactions maintain immune tolerance and regulate innate and adaptive immune responses. Microbial metabolites also promote regulatory T-cell differentiation and limit excessive inflammation. Loss of this homeostatic balance may contribute to systemic inflammation and CKD progression [26,27].

#### 3.1.2. Microbial Metabolites and Host Homeostasis

Short-chain fatty acids (SCFAs), including acetate, propionate, and butyrate, are produced through bacterial fermentation of dietary fiber. Butyrate is a major energy source for colonocytes and supports epithelial integrity and mucosal immunity. SCFAs also regulate inflammatory and metabolic pathways through G-protein-coupled receptors [28,29].

SCFAs inhibit NF-κB signaling, reduce proinflammatory cytokine production, and promote regulatory T-cell differentiation. They also strengthen tight junctions and reduce intestinal permeability. Reduced SCFA production may indicate impaired microbial function and intestinal barrier integrity. However, these changes are not specific to CKD and should be interpreted together with established clinical and renal biomarkers [27,29,30].

#### 3.1.3. Intestinal Barrier Integrity and Mucosal Immunity

The intestinal epithelial barrier includes epithelial cells, mucus, antimicrobial peptides, immune cells, and intercellular junctions. Tight junction proteins, including claudins, occludin, and zonula occludens proteins, regulate paracellular permeability and limit the passage of luminal antigens and microbial products into the circulation [16,31,32].

Barrier disruption increases the systemic translocation of lipopolysaccharides, bacterial DNA fragments, and microbial metabolites. These products activate Toll-like receptor-dependent pathways and promote oxidative stress and chronic inflammation. Measures of intestinal permeability and microbial translocation may therefore provide mechanistic information in CKD. Their diagnostic and prognostic value, however, requires further clinical validation (Figure 1) [16,33].

### 3.2. Gut Dysbiosis and Intestinal Barrier Dysfunction in CKD

CKD is associated with changes in the composition, diversity, and metabolic activity of the intestinal microbiota. Reduced microbial diversity, depletion of short-chain fatty acid (SCFA)-producing commensals, and enrichment of taxa involved in uremic toxin generation have been reported. These alterations appear more pronounced in advanced CKD and kidney failure, although their magnitude varies across patient populations and analytical methods [15,17,34].

Reported changes include reduced abundance of butyrate-producing genera such as *Faecalibacterium* and *Roseburia*, as well as other SCFA-associated or metabolically cross-feeding genera, including *Prevotella* and *Bifidobacterium*. In contrast, bacteria with urease, indole-forming, or p-cresol-forming activity may be enriched. Increased representation of *Enterobacteriaceae*, *Enterococcus*, *Clostridium*, and members of the phylum *Proteobacteria* has also been described. These functional shifts favor the production of ammonia and the microbial precursors of indoxyl sulfate, pCS, and trimethylamine N-oxide [35,36,37].

Uremia itself contributes to dysbiosis. Urea diffuses into the intestinal lumen, where bacterial urease generates ammonia and ammonium, increases luminal pH, and disrupts epithelial tight junctions. This creates a bidirectional cycle in which reduced renal clearance modifies the intestinal environment, while microbial products may amplify systemic inflammation and renal injury [16,37,38]. Microbiota composition is also influenced by reduced fiber intake, dietary restrictions, metabolic acidosis, intestinal edema, delayed transit, iron supplementation, phosphate binders, antibiotics, and dialysis-related factors. These variables may confound microbiome comparisons and limit the specificity of individual microbial signatures as CKD biomarkers [34,39,40].

Intestinal barrier dysfunction provides a mechanistic link between dysbiosis and systemic inflammation. Uremic conditions are associated with altered expression and organization of claudins, occludin, and zonula occludens-1. The resulting increase in paracellular permeability facilitates the passage of microbial products into the circulation [16,33,38,41]. Oxidative stress, intestinal ischemia, local inflammation, and ammonia-mediated epithelial injury contribute to this process. Depletion of butyrate-producing bacteria may further impair colonocyte metabolism and tight junction integrity [16,30,33,38].

Increased permeability promotes the systemic translocation of LPSs and other pathogen-associated molecular patterns. LPSs activate TLR4 and NF-κB signaling, inducing the transcription of proinflammatory cytokines, chemokines, and adhesion molecules. This pathway provides a priming signal for the NLRP3 inflammasome but does not, by itself, constitute direct inflammasome activation [18,33].

Elevated concentrations of C-reactive protein (CRP), interleukin-6 (IL-6), tumor necrosis factor-α (TNF-α), and monocyte chemoattractant protein-1 (MCP-1) have been reported in advanced CKD and kidney failure. Although CRP and IL-6 are associated with adverse CKD outcomes, these inflammatory markers are not specific to gut barrier dysfunction [42,43] (Figure 2). Microbial profiles, SCFA concentrations, intestinal permeability markers, circulating lipopolysaccharides, and inflammatory mediators should therefore be considered candidate research biomarkers. Their diagnostic and prognostic use requires standardized sampling, harmonized analytical methods, adjustment for renal function and treatment-related confounders, and validation beyond established measures such as eGFR and albuminuria.

### 3.3. Gut-Derived Uremic Toxins

IS, pCS, and trimethylamine N-oxide (TMAO) are microbiota-derived metabolites that accumulate as renal function declines. Their circulating concentrations reflect microbial precursor production, hepatic metabolism, and renal clearance. Higher concentrations have been associated with CKD progression and cardiovascular complications, although the strength of these associations varies according to the toxin, measured fraction, CKD stage, and patient population [44,45,46,47]. Their biogenesis, biological effects, clinical associations, and biomarker limitations are summarized in Table 1.

Despite their biological plausibility, IS, pCS, and TMAO cannot currently be considered established diagnostic biomarkers. Reduced renal clearance may account for part of their association with adverse outcomes, making it difficult to distinguish cause from consequence. An earlier meta-analysis reported substantial between-study heterogeneity in analyses of free pCS and evidence of publication bias for cardiovascular outcomes. After trim-and-fill adjustment, the association between free pCS and cardiovascular events was attenuated and was no longer statistically significant [62]. Clinical translation will require standardized sampling and analytical methods, separate reporting of total and free protein-bound fractions, adjustment for eGFR and dialysis-related factors, and prospective assessment of their incremental value beyond eGFR and albuminuria.

### 3.4. NLRP3 Inflammasome Priming and Activation

The NLRP3 inflammasome is a cytosolic complex composed of the NLRP3 sensor, the Apoptosis-associated Speck-like (ASC) adaptor protein and pro-caspase-1. Complex assembly activates caspase-1, which cleaves pro-IL-1β and pro-IL-18 into their mature forms and initiates gasdermin D-dependent pyroptosis [63,64].

NLRP3 signaling requires a distinction between priming and activation. Priming, or signal 1, is initiated by pattern-recognition receptors such as Toll-like receptors. Subsequent NF-κB signaling increases the transcription of NLRP3 and pro-IL-1β. Activation, or signal 2, promotes assembly of the NLRP3–ASC–pro-caspase-1 complex. Relevant triggers include reactive oxygen species (ROS), mitochondrial dysfunction, extracellular ATP, potassium efflux, lysosomal injury, and uric acid or calcium oxalate crystals [18,63,65].

CKD provides a biological environment that may facilitate both stages. Uremia, oxidative stress, endotoxemia, and mitochondrial injury can enhance inflammasome-related signaling. Increased expression of NLRP3 components has been reported in podocytes, mesangial cells, tubular epithelial cells, endothelial cells, and infiltrating macrophages [11,12]. However, increased NLRP3 expression indicates priming capacity and does not, by itself, demonstrate inflammasome assembly or caspase-1 activation.

NF-κB is central to inflammasome priming. Gut-derived LPSs activate TLR4 and NF-κB signaling, increasing the transcription of NLRP3, pro-IL-1β, and other inflammatory mediators. LPS exposure should therefore be interpreted primarily as evidence of signal 1 rather than direct inflammasome activation. ROS and mitochondrial dysfunction may further amplify NF-κB activity and inflammatory priming [18,33,66].

Indoxyl sulfate: Evidence for IS is cell-type-dependent. In proximal tubular cells, IS increased NLRP3 expression, caspase-1 activity, and IL-1β secretion through ROS- and IKK-dependent pathways. In macrophages, however, IS increased pro-IL-1β transcription through AhR–NF-κB/MAPK signaling while reducing NLRP3 expression and leaving caspase-1 activity unchanged. Reduced NLRP3 inducibility has also been reported in hemodialysis patients. Additional experiments linked IS to ROS production, tubular senescence, and low-grade inflammation without demonstrating NLRP3 activation [49]. Direct activation has mainly been reported in cardiovascular cell types. Overall, IS appears to act predominantly as a priming stimulus, while evidence for direct activation remains inconsistent [19,20,49,50,67].

p-Cresyl sulfate: pCS promotes ROS generation and inflammatory gene expression, creating conditions compatible with NLRP3 priming. Direct evidence of inflammasome assembly or caspase-1 activation is currently lacking [21,54].

Trimethylamine N-oxide: TMAO has the strongest preclinical evidence for signal 2 activation among the three metabolites, although this evidence derives entirely from animal and cell-based models rather than human tissue. In an animal study using nephrectomized CKD rats, TMAO increased renal NLRP3 formation, cleaved caspase-1, IL-1β, inflammatory infiltration, and oxidative stress. In an in vitro study using cultured human renal fibroblasts, TMAO promoted proliferation and collagen production through PERK–Akt–mTOR (PKR-like Endoplasmic Reticulum Kinase—Protein Kinase B—mechanistic Target of Rapamycin)–NLRP3 signaling. Preclinical vascular studies have also linked TMAO to NLRP3-dependent calcification and endothelial inflammation [57,58,59,60,68] (Figure 3).

Overall, the available evidence supports a graded relationship between gut-derived factors and NLRP3 signaling. LPSs are an established priming stimulus. IS acts predominantly through priming, although activation has been reported in selected cell types. pCS creates a proinflammatory environment but lacks direct activation evidence. TMAO has the strongest preclinical evidence for NLRP3 assembly and caspase-1 activation, but this evidence is confined to cell and animal models and has not been confirmed in human tissue. However, none of these pathways has been validated as a clinical diagnostic or prognostic marker.

### 3.5. Downstream Renal and Systemic Injury

Oxidative stress links gut-derived metabolites, inflammation, and structural renal injury. In CKD, mitochondrial dysfunction, NADPH oxidase activity, inflammatory cell infiltration, uremic toxins, and impaired antioxidant defenses increase reactive oxygen species (ROS) production. Persistent ROS generation promotes lipid, protein, and DNA damage, endothelial dysfunction, and profibrotic signaling [69,70]. Experimental studies suggest that IS, pCS, and TMAO can increase ROS generation in renal or vascular cells. ROS-dependent signaling can amplify NF-κB activity and facilitate NLRP3 activation, thereby linking metabolic stress with inflammation and fibrosis [70,71].

NLRP3 activation induces caspase-1-dependent pyroptosis, an inflammatory form of programmed cell death characterized by cellular swelling, membrane pore formation, and release of intracellular inflammatory mediators. Cleavage of gasdermin D is a central molecular event in this process [64,71].

Experimental studies have identified pyroptotic injury in tubular epithelial cells, podocytes, endothelial cells, and infiltrating immune cells. The resulting cytokine release promotes macrophage recruitment, oxidative stress, and fibroblast activation. Persistent inflammasome signaling may also enhance TGF-β and connective tissue growth factor expression, collagen synthesis, and extracellular matrix deposition [70,71].

Endothelial and podocyte injury represent important renal and systemic consequences of this inflammatory environment. Gut-derived toxins impair nitric oxide bioavailability and promote ROS generation, NF-κB signaling, leukocyte adhesion, and vascular inflammation [70,72]. Podocytes are particularly vulnerable because of their limited regenerative capacity and essential role in maintaining the glomerular filtration barrier. In experimental diabetic podocytes, IS aggravated ROS production and altered nephrin, synaptopodin, CD2-associated protein (CD2AP), and TGF-β1/Smad-related signaling. These findings support a preclinical podocyte-injury mechanism but do not establish the same effect in humans. These alterations increase glomerular permeability and contribute to albuminuria and glomerulosclerosis. The combined endothelial and podocyte injury may accelerate both CKD progression and cardiovascular complications [73].

TGF-β/Smad signaling is a major pathway connecting chronic inflammation to renal fibrosis. It promotes fibroblast activation, myofibroblast differentiation, extracellular matrix synthesis, and reduced matrix degradation. Experimental disruption of TGF-β1/Smad3 signaling attenuates collagen accumulation and tubulointerstitial fibrosis, supporting a causal role for this pathway in renal scarring. TGF-β and ROS form a reciprocal amplification loop, while IS may further enhance TGF-β-dependent extracellular matrix production [70,73,74].

Tubulointerstitial fibrosis is characterized by tubular atrophy, interstitial inflammation, capillary rarefaction, myofibroblast accumulation, and excessive deposition of collagen and fibronectin. Its severity is closely associated with loss of kidney function across different CKD etiologies. Tubular epithelial cells, fibroblasts, endothelial cells, macrophages, and pericytes contribute to this maladaptive repair response [70,74]. Within the gut–kidney axis, endotoxemia, uremic toxins, oxidative stress, and inflammasome signaling converge on NF-κB- and TGF-β-dependent pathways, providing a plausible mechanism for progressive renal remodeling [70,71].

Glomerulosclerosis develops through podocyte depletion, mesangial matrix expansion, glomerular basement membrane remodeling, and capillary collapse. Experimental podocyte loss induces proteinuria, glomerular scarring, and declining kidney function. Oxidative stress, inflammatory cytokines, uremic toxins, and TGF-β signaling may contribute to this process. Glomerulosclerosis and tubulointerstitial fibrosis reinforce each other through proteinuria, tubular inflammation, hypoxia, and nephron loss [75].

These downstream lesions are common to several CKD etiologies and are not specific to gut-derived injury. They may indicate disease activity or progression but cannot independently establish activation of the gut–toxin–NLRP3 pathway. Mechanistic biomarkers should therefore be interpreted together with established measures of glomerular and tubular injury.

### 3.6. Human Biomarker Evidence and Clinical Utility

Human studies have evaluated the gut–kidney–NLRP3 axis at the microbial, metabolite, and inflammatory levels. Gut microbial composition is assessed mainly by 16S rRNA gene sequencing or shotgun metagenomics. However, the results are sensitive to stool collection, DNA extraction, sequencing platform, and bioinformatic processing [76].

IS, pCS, and TMAO are generally measured in plasma or serum using high-performance liquid chromatography or targeted liquid chromatography–tandem mass spectrometry. For IS and pCS, total and free concentrations are not interchangeable because albumin binding substantially affects the circulating free fraction [77,78].

Cytokines are commonly quantified by immunoassays. Nevertheless, circulating IL-1β or IL-18 alone cannot confirm NLRP3 activation. More specific evidence requires markers such as ASC specks, cleaved caspase-1, or cleaved gasdermin D, which remain confined to research settings [71,79].

Current human evidence is predominantly prognostic rather than diagnostic. Cross-sectional microbiome and metabolomic studies identify group-level differences between patients with CKD and controls. Few studies, however, report diagnostic accuracy, calibration, external validation, or clinically applicable thresholds. Higher circulating IS and pCS concentrations, particularly their free fractions, have been associated with mortality and cardiovascular events. TMAO has also predicted mortality in selected CKD cohorts. These associations remain heterogeneous and do not establish these metabolites as diagnostic markers of CKD, dysbiosis, or renal inflammasome activation [47,62].

Reported discrimination for these markers is modest and outcome-dependent. For IS, a receiver operating characteristic (ROC) analysis for peripheral arterial stiffness in non-dialysis CKD stages 3–5 yielded an area under the curve (AUC) of 0.667 (95% CI 0.580–0.754), with the best cut-off of 0.996 μg/mL achieving 66.1% sensitivity and 60.7% specificity [80]; however, a separate prospective cohort of CKD patients with heart failure found no significant association between serum IS and one-year cardiovascular events, illustrating population-dependent heterogeneity.

By contrast, a prospective multicenter cohort of 300 patients with heart failure and CKD stages G3b–G5 found no association between serum IS and the one-year composite of all-cause death, hospitalization for heart failure, nonfatal myocardial infarction, or nonfatal stroke. The hazard ratio for the high- versus low-IS group was 1.00 (95% CI, 0.58–1.70), and the AUC was 0.53 (95% CI, 0.44–0.62), illustrating marked population- and outcome-dependent heterogeneity [45].

For serum total pCS, ROC analysis for aortic stiffness yielded an AUC of 0.667 (95% CI, 0.570–0.753), with an optimal cut-off of 8.39 mg/L providing 78.0% sensitivity and 57.39% specificity [55]. A pooled meta-analysis found free pCS to be associated with cardiovascular events (pooled OR, 1.28; 95% CI, 1.10–1.50), whereas free IS was not (pooled OR, 1.05; 95% CI, 0.98–1.13). However, the pCS analysis showed substantial heterogeneity (I^2^ = 80.7%; *p* < 0.001) and evidence of publication bias (Egger’s test *p* < 0.001); after trim-and-fill adjustment, the estimate was attenuated to 1.10 (95% CI, 0.93–1.27) [62].

A peritoneal dialysis cohort identified a ROC-derived cut-off of 18.99 mg/L for total pCS, predicting a composite of technique failure and cardiovascular events [81]. For TMAO, a prospective cohort of 521 CKD patients found that adding TMAO to a model of traditional risk factors, eGFR, and high-sensitivity CRP improved the AUC from 0.633 to 0.660 and yielded a net reclassification improvement of 17.3% for five-year all-cause mortality—the clearest evidence to date of incremental prognostic value beyond eGFR for any of the three metabolites, though it awaits external replication. Across all three toxins, no study has reported prospective external validation of a fixed decision threshold, and reported AUCs in the 0.63–0.67 range indicate weak-to-moderate discrimination rather than diagnostic-grade performance [47].

A more recent multicenter observational study of 272 participants reported an AUC of 0.916 for identifying reduced eGFR using plasma TMAO. However, this cross-sectional result primarily demonstrates concordance between TMAO accumulation and renal impairment; it does not establish longitudinal prognostic value or incremental clinical utility beyond eGFR [78].

Overall, biomarker performance is outcome-dependent. The IS and pCS vascular-stiffness studies demonstrated only modest discrimination, while the higher AUC reported for TMAO largely reflects its close relationship with renal clearance. None of the fixed thresholds described above has undergone prospective external validation for clinical decision-making.

Renal function is a major determinant of biomarker concentrations. Reduced glomerular filtration and tubular secretion directly increase circulating toxin levels. Therefore, an elevated concentration may reflect impaired clearance rather than increased intestinal production or upstream inflammasome activity. This distinction differs by toxin: IS and pCS are cleared predominantly by proximal tubular secretion of the protein-bound fraction, a pathway disproportionately impaired relative to glomerular filtration as CKD progresses, so their accumulation reflects both increased colonic production from dietary tryptophan and tyrosine substrate and impaired secretory clearance [44,48].

TMAO, in contrast, is a small, water-soluble molecule eliminated predominantly by the kidneys. Glomerular filtration appears to be the major route, although some clearance data suggest a contribution from tubular secretion, and the relative importance of these pathways remains uncertain. Consequently, circulating TMAO concentrations closely track declining eGFR, while hepatic flavin-containing monooxygenase 3 activity and dietary precursor intake (choline, carnitine, and betaine) independently modulate production and confound this relationship [47,56,82].

Albuminuria provides an established measure of renal injury and prognosis, while serum albumin influences the free fractions of IS and pCS. Biomarker studies should consequently adjust for both eGFR and urinary albumin-to-creatinine ratio. Although some associations remain significant after multivariable adjustment, incremental prognostic value beyond these conventional measures has not been consistently demonstrated [47,77].

Protein-bound uremic toxin clearance may provide complementary information on proximal tubular secretory function. Reduced IS and pCS clearance has been associated with urinary tubular injury markers independently of eGFR in diabetic kidney disease. These findings remain exploratory and require validation in larger, independent cohorts [48]. At present, the absence of harmonized assays, validated reference ranges, and standardized clinical thresholds prevents routine implementation. Future studies should determine whether these biomarkers improve discrimination, risk classification, or clinical decision-making beyond eGFR and albuminuria [76,77].

The analytical methods, potential clinical applications, and current validation gaps of candidate biomarkers of the gut–kidney–NLRP3 axis are summarized in Table 2.

### 3.7. Evidence from Interventional Studies

Human interventional evidence for the gut–kidney–NLRP3 axis remains heterogeneous. The available studies include disease-specific mucosal therapy, microbiome-directed interventions, intestinal toxin adsorption, modified dialysis techniques, and early-phase inflammasome inhibition. Most studies assessed changes in microbial composition, uremic toxin concentrations, or inflammatory markers. Kidney failure, cardiovascular events, and mortality were rarely evaluated. The principal completed and ongoing studies are summarized in Table 3.

These interventions can be grouped into three mechanistically distinct categories: (1) interventions that modify microbiome composition (probiotics, prebiotics, synbiotics, and fecal microbiota transplantation), (2) interventions that reduce toxin exposure or absorption without altering the microbiome (oral adsorbents such as AST-120, adsorptive hemodiafiltration, and dietary protein restriction), and (3) interventions that directly target inflammatory or NLRP3 signaling (selective NLRP3 inhibitors such as AZD4144). Most human studies across these categories have relied on surrogate endpoints, including uremic toxin concentrations, inflammatory biomarkers, or short-term eGFR changes. The long-term AST-120 trial evaluated clinical renal outcomes but did not demonstrate a significant clinical benefit. Overall, no completed randomized trial has yet shown a benefit for kidney failure, cardiovascular events, or mortality.

Evidence specific to the dialysis population, in whom gut-derived uremic toxins accumulate most severely, has expanded substantially. A systematic review and meta-analysis of 23 randomized controlled trials comprising 931 hemodialysis patients found that probiotic, prebiotic, and synbiotic supplementation significantly reduced circulating pCS, endotoxin, malondialdehyde, C-reactive protein, and interleukin-6, alongside improvements in total antioxidant capacity [97].

Consistent with this, a network meta-analysis pooling 331 patients across CKD stages 3a–5 ranked microbiota-targeted interventions by SUCRA score and found the largest effects for IS reduction (SUCRA 88.5% for the free fraction, 83.1% for total), with more modest rankings for pCS (72.6% free, 66.2% total) [98].

Animal studies provide complementary mechanistic support but do not uniformly align with these clinical findings. In a rat model, IS administration directly activated the NLRP3 inflammasome—upregulating NLRP3, ASC, and procaspase-1 and increasing IL-1β/IL-18—driving cardiac fibrosis and contractile dysfunction [50].

This contrasts with human and ex vivo data: IS has been shown to promote macrophage IL-1β production via AhR/NF-κB/MAPK signaling without activating the NLRP3 inflammasome itself [20], and hemodialysis patients’ peripheral blood mononuclear cells show markedly reduced NLRP3 inflammasome inducibility upon canonical stimulation, with IS pretreatment further blunting subsequent inflammasome activation [67]. This animal–human discrepancy may reflect differences in toxin exposure duration and dose (acute, high-dose rodent protocols versus chronic, lower-grade human exposure, which may induce compensatory immune tolerance), species-specific differences in aryl hydrocarbon receptor signaling, and the single-toxin experimental design of most animal studies compared with the multi-toxin uremic milieu in humans. This gap underscores the need for future dialysis-population trials that pair microbiome-targeted interventions with inflammasome-related biomarkers and, where feasible, activation-specific markers such as ASC speck formation, cleaved caspase-1, mature IL-1β and IL-18, or cleaved gasdermin D, rather than relying on circulating uremic toxin concentrations alone.

Human evidence linking sodium–glucose cotransporter-2 inhibitors to the gut–kidney axis is less direct. A matched case–control study of 60 patients with predialysis CKD and 30 non-CKD controls found lower concentrations of IS, pCS, and several SCFAs in SGLT2 inhibitor-treated patients. Distinct microbial profiles were also identified. However, the observational design precluded causal inference [99]. In a 12-week randomized study in patients with type 2 diabetes, neither dapagliflozin nor gliclazide produced a significant change in fecal microbiome composition. No CKD trial has established that the renal effects of SGLT2 inhibition are mediated by microbiome modification [100].

Dietary, prebiotic, probiotic, and synbiotic studies have produced variable results. The SYNERGY trial reduced serum pCS but not its primary IS endpoint [83]. NATUREN G reduced free IS without changing eGFR or CRP [84]. ProLowCKD showed a favorable trend in IS among patients receiving probiotics with a low-protein diet [85]. In contrast, the 12-month SYNERGY II feasibility trial found no reduction in IS or pCS and recorded a decline in eGFR in both study groups [86]. A small randomized trial by Mitrović et al. reported reductions in IS and hsCRP and a short-term increase in eGFR. A subsequent 84-patient conference report from the same group found reductions in IS, pCS, and hsCRP but did not report an eGFR outcome [87,88]. A meta-analysis of 21 studies identified modest reductions in blood urea nitrogen and CRP, without significant effects on eGFR or serum creatinine. Considerable heterogeneity was present in formulations, doses, populations, and follow-up periods [101]. A network meta-analysis of 53 trials further suggested that combined multi-strain probiotic regimens outperform single- or dual-strain preparations for improving renal function and metabolic and inflammatory markers in CKD. Controlled CKD evidence for glucomannan, direct SCFA supplementation, and fecal microbiota transplantation remains limited [102].

Toxin-directed interventions have also yielded inconsistent findings. In a 579-patient randomized trial, 36 months of AST-120 did not improve the composite renal outcome, eGFR decline, mortality, hospitalization, or quality of life. Serum concentrations of the measured uremic toxins were not significantly reduced [91]. In a separate randomized open-label study of patients receiving maintenance hemodialysis, 4 weeks of AST-120 reduced pruritus scores and produced transient reductions in IS, pCS, and TNF-α. The study did not assess long-term renal or cardiovascular outcomes [92].

Conventional dialysis removes protein-bound toxins inefficiently. A sequential feasibility study involving 20 hemodialysis patients found that adsorptive polymethylmethacrylate hemodiafiltration reduced IS and inflammatory markers over 6 months but did not reduce pCS. The uncontrolled sequential design and small sample precluded evaluation of clinical efficacy [93]. Albumin-binding displacement, hemoperfusion, albumin-enriched dialysate, and combined displacement–adsorption systems have mainly been examined in technical, preclinical, or short-term clearance studies [44,103,104].

Direct NLRP3 inhibition has only recently entered CKD-focused clinical development. Phase 1 studies of AZD4144 provided initial safety, pharmacokinetic, and target-engagement data. A randomized phase 1b study enrolled 29 participants with atherosclerotic cardiovascular disease and CKD, while a separate pharmacokinetic study enrolled 41 participants with severe renal impairment, kidney failure, or normal renal function. Both studies have been completed, but clinical outcome results are not yet available. These trials were designed primarily to evaluate safety, pharmacokinetics, and short-term inflammatory biomarkers rather than CKD progression [94,95,96,105].

Overall, interventional studies demonstrate that several approaches can modify microbial composition, circulating toxin concentrations, or inflammatory biomarkers. These effects have not translated consistently into improvements in eGFR, albuminuria, kidney failure, cardiovascular events, or mortality. Targeted-release budesonide represents an exception with demonstrated renal efficacy, but its evidence is restricted to IgA nephropathy and does not validate the broader gut–toxin–NLRP3 pathway.

## 4. Discussion

### 4.1. Principal Findings

The principal finding of this review is that the evidence supporting inflammasome priming is substantially stronger than that supporting direct NLRP3 activation by gut-derived uremic toxins. CKD-associated dysbiosis and intestinal barrier dysfunction increase systemic exposure to LPSs and pro-oxidant microbial metabolites [33]. LPS-mediated TLR4/NF-κB signaling provides a well-established priming signal by increasing the transcription of NLRP3 and pro-IL-1β [18]. IS and pCS may further promote this inflammatory state through reactive oxygen species generation and NF-κB activation [20,54].

Evidence for the activation step is less consistent and differs among toxins. IS has been associated with caspase-1 activation and IL-1β release in renal tubular cells. However, macrophage studies predominantly indicate enhanced priming without consistent NLRP3 assembly or activation. Reduced inflammasome responsiveness has also been reported in patients undergoing hemodialysis, supporting a cell- and context-dependent effect [19,20,67]. For pCS, current evidence demonstrates oxidative and inflammatory signaling, but direct activation of the NLRP3 inflammasome has not been established [21,54]. In contrast, TMAO has the most consistent evidence for activation. Increased NLRP3 expression, caspase-1 activity, and mature IL-1β production have been demonstrated in experimental CKD, renal fibroblast, podocyte, and vascular models. Nevertheless, these findings remain predominantly preclinical [58,60,68].

The distinction between priming and activation is important for biomarker interpretation. Increased NLRP3 expression, NF-κB activity, or circulating IL-1β and IL-18 concentrations do not independently demonstrate formation of an active inflammasome. More specific evidence requires assessment of ASC oligomerization or speck formation, caspase-1 cleavage, mature cytokine release, or gasdermin D cleavage, ideally combined with selective NLRP3 inhibition [63,64]. Most human studies have measured circulating toxin concentrations and nonspecific inflammatory markers rather than these activation-specific endpoints. Their interpretation is further limited by renal clearance, eGFR, albuminuria, and the absence of standardized biomarker thresholds [48,106].

Overall, the available evidence supports a sequential model in which gut dysbiosis, barrier dysfunction, and endotoxemia establish inflammasome priming, whereas direct activation requires additional cellular stress signals. Among the evaluated metabolites, TMAO is the most consistently supported candidate for providing such an activation signal. However, no clinical study has yet demonstrated that reducing gut-derived toxin exposure suppresses NLRP3 activation and improves renal outcomes. The gut–toxin–inflammasome axis should therefore be considered mechanistically plausible and partially demonstrated, but not yet validated as a diagnostic, prognostic, or therapeutic framework [83,91,92,93,94,95,96,105].

### 4.2. Diagnostic and Prognostic Implications

Several components of the gut–kidney–inflammasome axis may be considered candidate biomarkers. Circulating total and free IS and pCS, as well as TMAO, reflect the combined effects of microbial metabolism and renal elimination. The clearance of protein-bound uremic toxins may also provide information on tubular secretory function that is not fully captured by glomerular filtration markers [48,107,108,109,110]. Upstream candidates include microbiome composition, microbial diversity, short-chain fatty acids, lipopolysaccharides, and markers of intestinal permeability. Downstream candidates include IL-1β, IL-18, caspase-1 activity, ASC oligomerization, and gasdermin D cleavage. The latter markers are mechanistically closer to inflammasome activation but are predominantly restricted to experimental assays [16,63,64,111,112,113,114,115,116,117,118,119,120].

Several factors currently prevent their implementation in routine diagnostics. Uremic toxin concentrations are influenced by diet, microbial production, intestinal transit, hepatic metabolism, albumin binding, tubular secretion, residual kidney function, and dialysis modality. Measurements of total and free IS or pCS are not interchangeable. Microbiome results are similarly affected by specimen collection, sequencing platform, analytical pipeline, medication exposure, and geographical variation. Standardized assays, reference intervals, clinically relevant thresholds, and validated sampling protocols remain unavailable for most proposed markers [40,76,110,111,112,121,122]. Circulating IL-1β and IL-18 are also nonspecific indicators of inflammation and do not independently demonstrate NLRP3 inflammasome activation [115,117,118,119,120,123].

Elevated IS, pCS, and TMAO concentrations have been associated with CKD progression, cardiovascular events, and mortality [42,45,124]. However, these associations are difficult to separate from reduced renal clearance. eGFR is a major determinant of toxin accumulation, while albumin concentration and albuminuria may affect the interpretation of protein-bound fractions. Although some associations persist after multivariable adjustment, few studies have demonstrated improved discrimination, calibration, or risk reclassification when these biomarkers are added to models containing eGFR and urinary albumin-to-creatinine ratio (UACR) [62,107,108,109,110,125,126,127]. Their incremental clinical value therefore remains uncertain.

At present, these biomarkers appear more suitable for prognostic research than for CKD diagnosis. IS, free pCS, and TMAO are the most developed prognostic candidates, whereas microbiome profiles and inflammasome-specific markers remain exploratory. Serial toxin measurements could potentially be used to monitor dietary, microbiome-directed, sorbent, or dialysis-based interventions. However, treatment-related changes in toxin concentrations have not been validated as surrogate endpoints for renal or cardiovascular outcomes [83,91,92,93]. Accordingly, these markers may complement eGFR and UACR in research settings, but they cannot currently replace established measures of kidney function and injury or guide individual clinical decisions.

### 4.3. Therapeutic and Biomarker-Monitoring Implications

The therapeutic implications of the gut–kidney–inflammasome axis should be interpreted according to the biological target of each intervention. Established treatments should continue to be monitored using validated renal endpoints. In IgA nephropathy, the response to targeted-release budesonide is assessed primarily based on proteinuria and eGFR rather than microbiome-derived markers. Similarly, the renal benefits of sodium–glucose cotransporter-2 inhibitors cannot currently be attributed to changes in the gut microbiota. Any associated changes in microbial composition or uremic toxin concentrations should therefore be considered exploratory [4,5,6,7,8,9,99,100,128,129,130].

Dietary interventions, prebiotics, probiotics, and synbiotics primarily target microbial composition and toxin generation. Their biological effects could be monitored using total and free IS and pCS, TMAO, short-chain fatty acids, and selected microbial signatures. Intestinal permeability and systemic inflammatory markers may provide complementary information. However, clinical studies have reported modest and inconsistent changes in these endpoints [83,84,85,86,89,101,131,132,133,134,135]. Because toxin concentrations are strongly influenced by renal clearance, serial measurements should be interpreted together with eGFR, UACR, serum albumin, dietary intake, and medication exposure [48,106].

For AST-120 and dialysis-based strategies, toxin measurements represent the most direct indicators of target engagement. Assessment should distinguish between total and free IS and pCS and, in dialysis studies, should account for pre- and post-dialysis concentrations, clearance, and post-dialysis rebound. Reductions in toxin concentrations or inflammatory markers may confirm a biological effect. Nevertheless, such reductions have not consistently translated into slower CKD progression or improved cardiovascular outcomes [91,92,93,104]. These measurements should therefore remain pharmacodynamic research endpoints rather than validated surrogate outcomes.

Monitoring direct NLRP3 inhibition requires more pathway-specific markers. CRP, IL-6, IL-1β, and IL-18 reflect systemic inflammation but do not independently establish inflammasome inhibition. More specific assessment could include ASC oligomerization or speck formation, cleaved caspase-1, mature IL-1β and IL-18, and gasdermin D cleavage. These markers should ideally be evaluated together with upstream toxin exposure and conventional renal outcomes [63,64,117,118,120]. Early clinical studies of AZD4144 may help define a pharmacodynamic signature of NLRP3 inhibition, but clinical validation in CKD is still required [94,95,96,105].

Overall, biomarker monitoring may help demonstrate target engagement and clarify biological response in clinical trials. It should not currently guide the initiation, modification, or discontinuation of treatment. Future studies should use standardized serial sampling and test whether biomarker changes provide information beyond eGFR and UACR and whether they predict clinically meaningful renal or cardiovascular outcomes.

The framework presented here, while centered on the gut–kidney axis, should be situated within a broader multiorgan network rather than treated as an isolated dyad. An integrated overview of mechanistic, translational, and clinical data implicates the gut microbiota as a key pathogenic node not only for the kidney but also for systemic disease more broadly, with CKD-associated dysbiosis, marked by depletion of short-chain-fatty-acid-producing taxa, potentially influencing several organs simultaneously [10,136].

The gut–liver connection is integral to the biogenesis of gut-derived uremic toxins: intestinally generated indole and p-cresol undergo hepatic conversion to IS and pCS, respectively, while trimethylamine is oxidized in the liver to TMAO [137,138]. Through the gut–cardiovascular pathway, these circulating metabolites and other microbial products may promote endothelial dysfunction, vascular inflammation, atherosclerosis, and adverse cardiovascular outcomes, although the available human evidence remains predominantly associative [42,124,136].

Direct organ–organ crosstalk further compounds this picture: nonalcoholic fatty liver disease independently increases the risk of CKD and accelerates its progression through shared metabolic and inflammatory pathways, linking hepatic dysfunction to renal impairment and worse cardiovascular outcomes largely independently of the gut compartment [139].

Cardiorenal crosstalk is also bidirectional: renal dysfunction promotes the accumulation of protein-bound uremic toxins with potentially harmful cardiac effects, while cardiac and renal dysfunction may mutually accelerate one another [136,140].

Future conceptual models of CKD progression may therefore benefit from framing the gut–kidney–NLRP3 axis as one node within a wider gut–liver–kidney–heart network, in which uremic toxins, dysbiosis, and inflammasome activation interact with direct hepatorenal and cardiorenal pathways to shape disease trajectory.

### 4.4. Limitations

#### 4.4.1. Limitations of the Evidence Base

The evidence underlying the gut–kidney–inflammasome axis is heterogeneous. Many mechanistic findings originate from animal models, isolated cell systems, or small observational cohorts. These models do not fully reproduce the chronic, multifactorial exposure characteristic of human CKD. Clinical studies also include diverse CKD etiologies, disease stages, dialysis modalities, dietary patterns, and medication exposures, limiting comparability and generalizability [40,71,114,141].

Microbiome studies are affected by differences in specimen collection, sequencing platforms, bioinformatic pipelines, geographical background, and taxonomic resolution. Similar limitations apply to metabolomic measurements. In particular, total and free concentrations of protein-bound toxins are not interchangeable, and standardized assays or clinically validated thresholds are lacking [40,48,76,106]. Reverse causation remains an important concern because declining renal clearance itself promotes toxin accumulation and intestinal dysbiosis. Associations between toxin concentrations and clinical outcomes may therefore reflect both microbial production and the severity of renal dysfunction [42,45,109].

Evidence linking individual toxins to NLRP3 activation is also uneven. IS produces cell-dependent effects, while pCS has not been shown to directly activate inflammasome assembly. TMAO has more consistent activation evidence, but this is derived predominantly from experimental models. Several studies used single-cell systems, short exposure periods, or toxin concentrations that may not represent chronic human exposure [19,20,49,50,54,58,60,67,71]. Moreover, increased NLRP3 expression or circulating IL-1β and IL-18 does not independently confirm activation of the assembled inflammasome [63,123].

The interventional literature is limited by small sample sizes, short follow-up, heterogeneous treatments, and inconsistent endpoints. Most studies assessed microbiome composition, toxin concentrations, inflammatory markers, or feasibility rather than kidney failure, cardiovascular events, or mortality. Consequently, biomarker improvement cannot yet be considered evidence of clinical benefit or accepted as a validated surrogate outcome [86,91,98,101,104,135].

#### 4.4.2. Limitations of the Review Methodology

This article is a narrative review and was not designed to meet the methodological requirements of a systematic review. The search was restricted to English-language publications indexed in PubMed/MEDLINE, Scopus, and Web of Science between January 2000 and September 2026, supplemented by manual reference screening. Relevant studies indexed elsewhere, published in other languages, or published before the prespecified period may therefore have been missed.

Study selection was based on author consensus and mechanistic or clinical relevance. Formal duplicate screening and data extraction were not performed. No prospectively registered review protocol, formal risk-of-bias assessment, certainty-of-evidence grading, or quantitative meta-analysis was used. These features introduce a risk of selection bias and prevent a formal comparison of effect sizes across studies.

The review integrates preclinical experiments, observational cohorts, clinical trials, systematic reviews, and trial registrations. This broad approach supports mechanistic interpretation but combines evidence from different methodological levels. It may also result in overlap between primary studies and evidence syntheses. In addition, results from ongoing or recently completed trials were not always available, limiting assessment of emerging interventions.

Finally, the review deliberately focuses on IS, pCS, TMAO, and NLRP3 signaling. Other microbial metabolites, immune pathways, and CKD-specific mechanisms were not examined comprehensively. The conclusions should therefore be interpreted as a focused qualitative synthesis rather than an exhaustive or quantitatively graded assessment of the gut–kidney axis.

### 4.5. Future Research Priorities

Future research should move beyond cross-sectional associations toward large, multicenter prospective cohorts. These studies should include patients across CKD stages, kidney disease etiologies, and dialysis modalities. Serial sampling is required to characterize within-patient variability and determine whether changes in IS, pCS, TMAO, microbiome profiles, or inflammasome-related markers precede CKD progression. Prespecified outcomes should include eGFR slope, sustained eGFR decline, kidney failure, cardiovascular events, and mortality. Analyses should account for eGFR, UACR, serum albumin, diet, medication exposure, comorbidities, residual kidney function, and dialysis modality. This approach may help distinguish microbial production from toxin accumulation caused by reduced renal clearance [42,48,106,109].

Standardization of pre-analytical and analytical procedures is essential. Sampling protocols should specify fasting status, recent dietary intake, specimen type, collection time, processing delay, storage conditions, and freeze–thaw exposure. Dialysis studies should standardize sampling relative to the dialysis session. Assay harmonization should address calibration, analytical units, quality-control materials, and the separate measurement of total and free protein-bound toxin fractions. Microbiome studies require comparable collection procedures, sequencing platforms, taxonomic resolution, and bioinformatic pipelines [40,76,106]. Inflammasome studies should also distinguish markers of priming from activation-specific endpoints. NLRP3 expression and circulating cytokine concentrations should not be considered equivalent to ASC assembly, caspase-1 activation, mature IL-1β or IL-18 release, or gasdermin D cleavage [63,64].

Candidate biomarkers and multivariable signatures should undergo external validation in independent populations. Validation cohorts should represent different geographical regions, ethnic groups, CKD etiologies, and stages of renal impairment. Thresholds and analytical procedures should be prespecified rather than optimized retrospectively in each dataset. Demonstrating reproducibility across laboratories and clinical populations will be necessary before these markers can be considered for routine use [142].

Multimarker frameworks integrating inflammatory, tissue-remodeling, oxidative, and epigenetic signals have been proposed in other heterogeneous diseases. Their potential clinical value depends on standardized measurement, disease-specific model development, and external validation rather than on the performance of individual biomarkers alone [142,143,144].

Most importantly, future studies must evaluate whether candidate biomarkers provide clinically relevant information beyond eGFR and UACR. Biomarker models should be compared with well-calibrated clinical reference models using measures of discrimination, calibration, risk reclassification, and decision-curve analysis. Statistical significance alone is insufficient. A marker will have clinical value only if it improves risk stratification, alters management, or identifies patients more likely to benefit from a specific intervention [142].

Interventional studies should incorporate serial toxin and inflammasome measurements alongside established renal and cardiovascular outcomes. They should determine whether biomarker changes mediate or predict treatment effects rather than merely demonstrate target engagement. This design would clarify whether these measurements can evolve from exploratory pharmacodynamic endpoints into validated prognostic or treatment-monitoring biomarkers [83,91,92,93,94,95,96,105].

## 5. Conclusions

The gut–kidney–inflammasome axis links intestinal dysbiosis and uremic toxin accumulation to inflammation and CKD progression. Evidence for inflammasome priming is substantially stronger than that for direct NLRP3 activation. IS acts mainly as a priming stimulus, direct activation by pCS remains unproven, and TMAO has the strongest experimental evidence for NLRP3 activation.

These metabolites and inflammatory mediators remain research biomarkers. Their routine use is limited by methodological heterogeneity, absent standardized thresholds, and uncertain added value beyond eGFR and UACR. Prospective studies with harmonized assays and external validation are required before their clinical implementation.

## Figures and Tables

**Figure 1:**
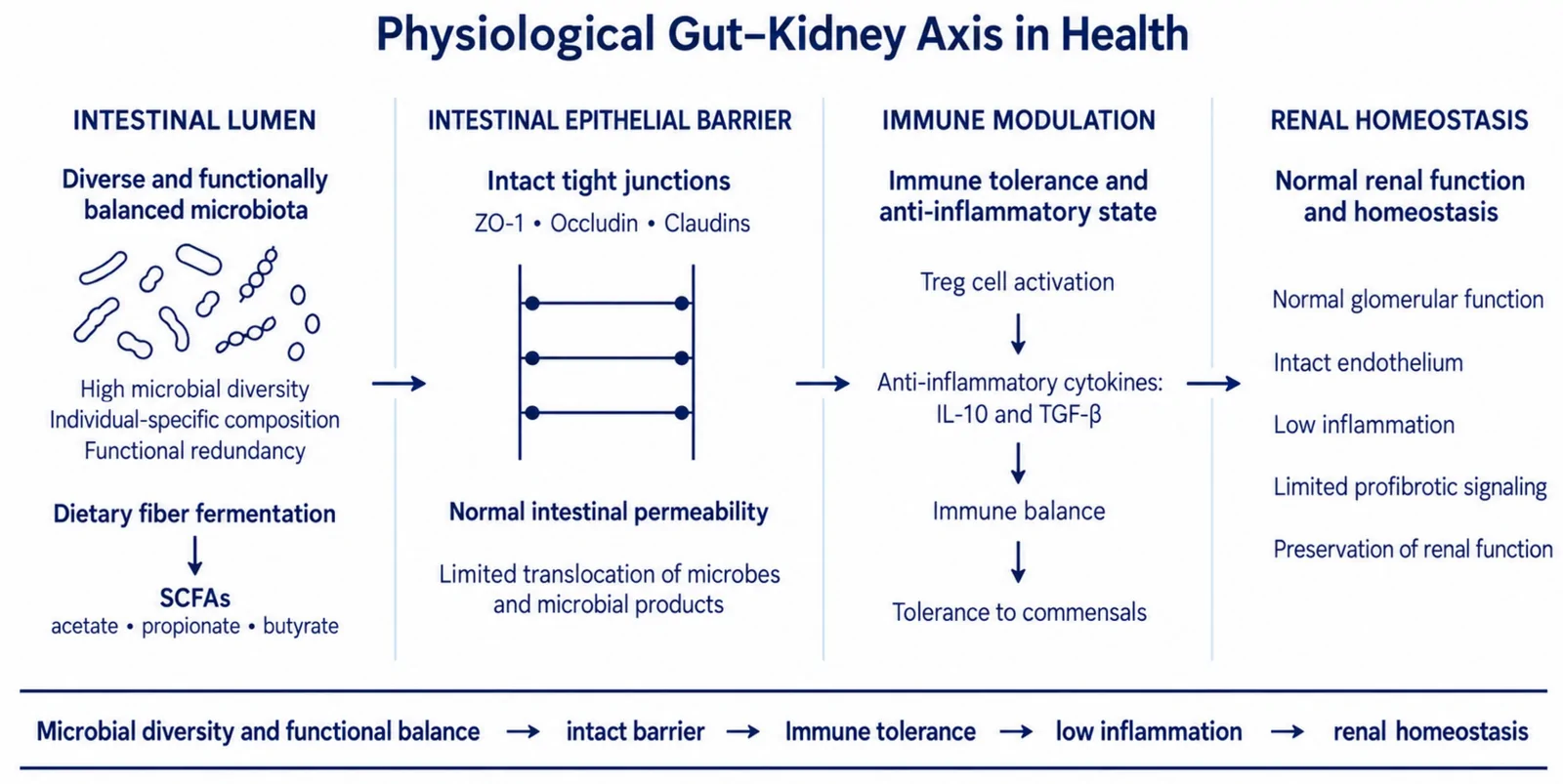
Physiological interactions between the intestinal microbiota, epithelial barrier integrity, immune modulation, and renal homeostasis. A physiologically diverse gut microbiota varies substantially among individuals but commonly includes SCFA-producing or metabolically cross-feeding genera such as *Faecalibacterium*, *Roseburia*, *Bifidobacterium*, and *Lactobacillus*, which contribute to the fermentation of dietary fiber into acetate, propionate, and butyrate. SCFAs reinforce epithelial tight junctions—zonula occludens-1 (ZO-1), occludin, and claudins—limiting intestinal permeability and translocation of endotoxins and bacterial products. SCFAs also promote regulatory T-cell (Treg) differentiation and anti-inflammatory cytokine production, including interleukin-10 (IL-10) and transforming growth factor beta (TGF-β). Together these interactions preserve endothelial function, restrain oxidative stress, maintain immune homeostasis, and support normal renal structure and function.

**Figure 2:**
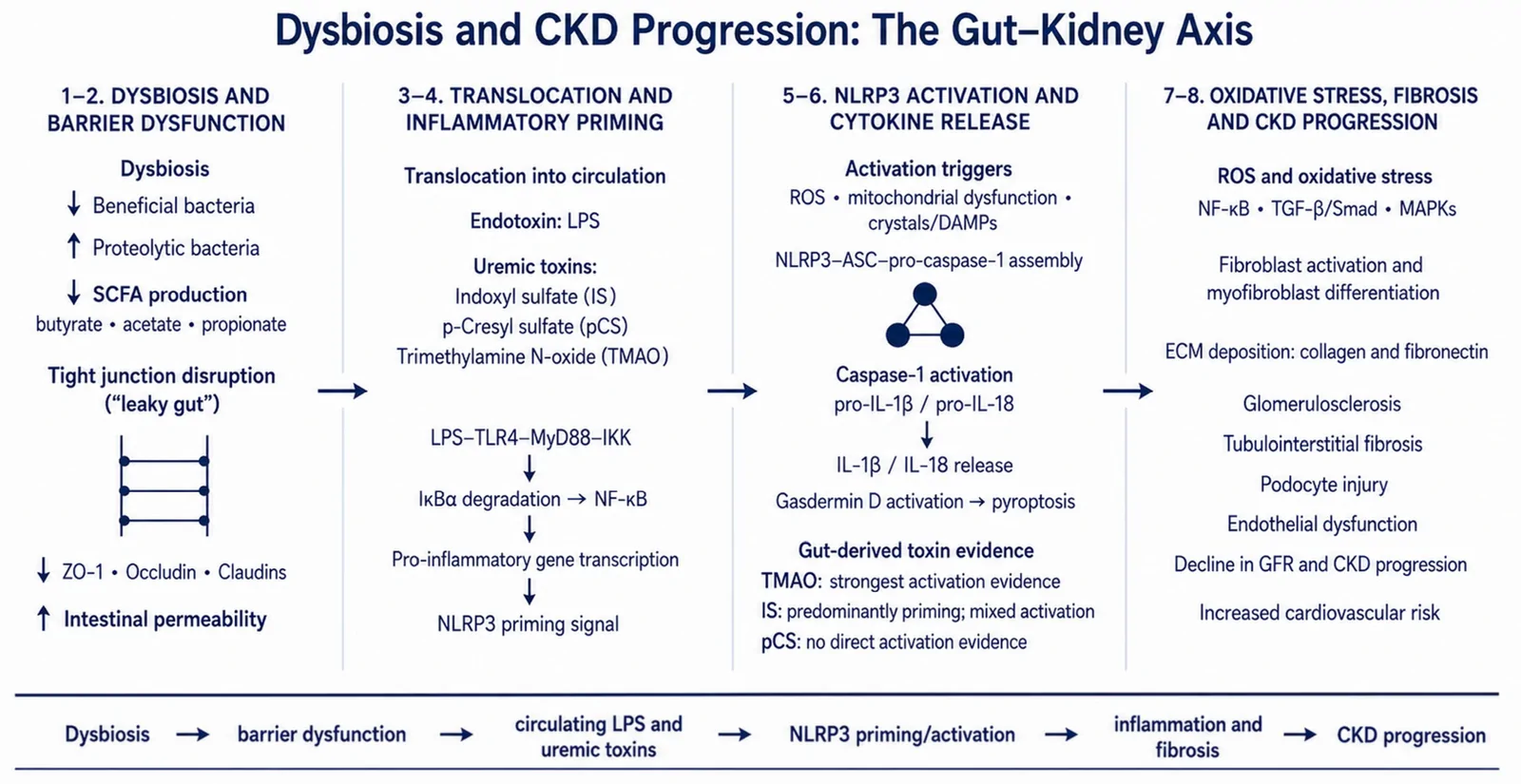
Proposed mechanisms linking intestinal dysbiosis to chronic kidney disease progression. Dysbiosis in CKD combines depletion of SCFA-producing bacteria with expansion of proteolytic, toxin-producing populations. Reduced SCFA availability disrupts tight junction proteins (ZO-1, occludin, claudins), increasing intestinal permeability (“leaky gut”) and allowing translocation of LPSs, bacterial products, and gut-derived uremic toxins—IS, pCS, and trimethylamine N-oxide (TMAO)—into the systemic circulation. These activate Toll-like receptor 4 (TLR4) and nuclear factor kappa B (NF-κB) signaling, priming transcription of proinflammatory mediators and inflammasome components. Subsequent NLRP3 activation drives caspase-1-dependent maturation of interleukin-1β (IL-1β) and IL-18, sustaining oxidative stress, pyroptosis, endothelial dysfunction, and fibrotic remodeling, and ultimately promoting glomerulosclerosis, tubulointerstitial fibrosis, declining glomerular filtration rate, and cardiovascular risk.

**Figure 3:**
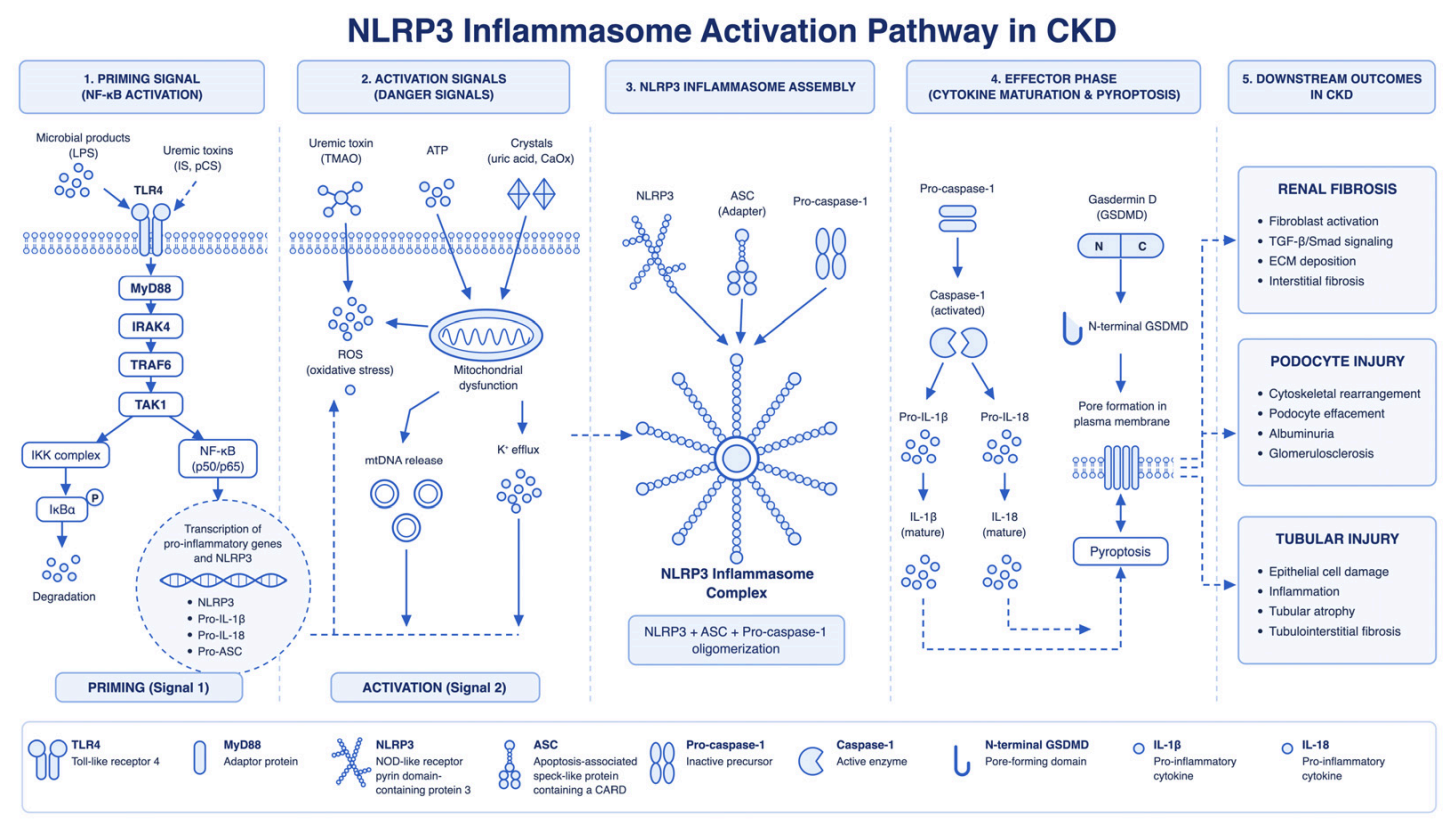
Molecular mechanisms of NLRP3 inflammasome activation and downstream inflammatory signaling in chronic kidney disease.

**Table 1:** Gut-derived uremic toxins in chronic kidney disease: mechanisms, clinical associations, and biomarker limitations.

Toxin	Biogenesis and Elimination	Molecular Effects	Renal and Cardiovascular Association	Limitations
Indoxyl sulfate (IS)	Dietary tryptophan is converted by bacterial tryptophanase to indole, followed by hepatic oxidation and sulfation. IS is strongly albumin-bound and is eliminated mainly by renal tubular secretion [44,48].	ROS generation; NF-κB, TGF-β, and RAAS signaling; mitochondrial dysfunction; endothelial injury; profibrotic signaling. IS appears to act mainly as an NLRP3-priming stimulus, whereas evidence for direct activation is cell-type-dependent and inconsistent [19,20,49,50].	Tubular epithelial injury; epithelial-to-mesenchymal transition; extracellular matrix deposition; tubulointerstitial fibrosis; vascular calcification; cardiovascular events and mortality [45,50].	Concentrations are strongly influenced by eGFR, tubular secretion, serum albumin, and dialysis modality. As a protein-bound solute, IS is poorly removed by conventional hemodialysis, while peritoneal clearance is also limited; in patients receiving peritoneal dialysis, residual renal function contributes substantially to total IS removal. High-flux, adsorptive, or extended-duration modalities achieve modestly greater clearance. Total and free IS fractions are not interchangeable. No validated clinical threshold or incremental diagnostic value has been established [51,52,53].
p-Cresyl sulfate (pCS)	Tyrosine and phenylalanine are fermented to p-cresol, which undergoes hepatic sulfation. pCS is strongly albumin-bound and is cleared predominantly through tubular secretion [44].	Oxidative stress; inflammatory signaling; leukocyte activation; endothelial dysfunction; tubular toxicity; profibrotic signaling. Direct activation of the NLRP3 inflammasome has not been demonstrated [21,54].	Interstitial inflammation; tubular injury; CKD progression; vascular dysfunction; hospitalization; cardiovascular morbidity and all-cause mortality [46].	Interpretation depends on eGFR, albumin binding, residual kidney function, and measurement of the total or free fraction. Being protein-bound, pCS is poorly cleared by either conventional hemodialysis or peritoneal dialysis. Residual renal function is a major determinant of total pCS removal in patients receiving peritoneal dialysis. Standardized clinical cutoffs are unavailable [51,52,53,55].
Trimethylamine N-oxide (TMAO)	Dietary choline, phosphatidylcholine, and L-carnitine are converted by intestinal bacteria to trimethylamine, followed by hepatic oxidation through flavin-containing monooxygenases. TMAO is eliminated primarily by the kidneys [47,56].	ROS production; NF-κB signaling; endothelial dysfunction; vascular inflammation; TGF-β/Smad-mediated fibrosis. Among the three metabolites, TMAO has the strongest experimental evidence for NLRP3 inflammasome activation [57,58,59,60].	Tubulointerstitial fibrosis; inflammatory renal injury; decline in kidney function; atherosclerosis; cardiovascular events; heart failure progression and mortality [47,57].	Concentrations are affected by renal clearance, diet, microbiota composition, and hepatic metabolism. Unlike IS and pCS, TMAO is not protein-bound and is more readily removed by conventional hemodialysis, although levels rebound between sessions owing to ongoing intestinal production; clearance by peritoneal dialysis is comparatively lower. Its causal contribution remains uncertain, and no CKD-specific diagnostic or prognostic threshold has been validated [56,61].

Abbreviations: CKD, chronic kidney disease; eGFR, estimated glomerular filtration rate; IS, indoxyl sulfate; NF-κB, nuclear factor kappa B; NLRP3, NOD-like receptor pyrin domain-containing protein 3; pCS, p-cresyl sulfate; RAAS, renin–angiotensin–aldosterone system; ROS, reactive oxygen species; TGF-β, transforming growth factor beta; TMAO, trimethylamine N-oxide.

**Table 2:** Analytical and clinical validation of candidate biomarkers of the gut–kidney–NLRP3 axis.

Biomarker Domain	Specimen and Analytical Method	Essential Reporting Requirements	Potential Clinical Application	Validation Gap
Gut microbiome profile [76]	Stool; 16S rRNA sequencing or shotgun metagenomics	Sampling protocol, DNA extraction, sequencing platform, bioinformatic pipeline	Patient stratification and monitoring of microbiome-directed interventions	No standardized CKD signature or validated diagnostic threshold
Circulating IS, pCS, and TMAO [47,55,77,78,80,82]	Serum or plasma; HPLC or targeted LC–MS/MS	Fasting status, total versus free toxin fraction, serum albumin, CKD stage, dialysis timing	Assessment of gut-derived metabolite burden and exploratory risk stratification	Strong dependence on renal clearance; no validated cutoffs or consistent incremental value beyond eGFR and albuminuria
Protein-bound uremic toxin clearance [48]	Paired plasma and timed urine samples; targeted LC–MS/MS	Urine collection interval, plasma concentration, eGFR, medication exposure	Assessment of proximal tubular secretory function	Evidence remains exploratory and is based mainly on a small diabetic kidney disease cohort
Circulating inflammatory markers [70,71]	Serum or plasma; immunoassays	Infection status, dialysis timing, comorbid inflammatory disease, renal function	General prognostic assessment and treatment-response monitoring	CRP, IL-6, IL-1β, and IL-18 are not specific to the gut–kidney axis or NLRP3 activation
Direct inflammasome activation markers [64,71,79]	Blood cells or renal tissue; ASC specks, cleaved caspase-1, mature IL-1β/IL-18, or cleaved gasdermin D	Cell population, sample processing, assay platform, distinction between expression and cleavage	Pathway-specific confirmation and pharmacodynamic monitoring	Limited human CKD data; no harmonized assay or clinical threshold

Abbreviations: ASC, apoptosis-associated speck-like protein containing a caspase recruitment domain; CKD, chronic kidney disease; CRP, C-reactive protein; eGFR, estimated glomerular filtration rate; HPLC, high-performance liquid chromatography; IL, interleukin; IS, indoxyl sulfate; LC–MS/MS, liquid chromatography–tandem mass spectrometry; NLRP3, NOD-like receptor pyrin domain-containing protein 3; pCS, p-cresyl sulfate; TMAO, trimethylamine N-oxide. Note: For IS and pCS, total and free concentrations should be reported separately. Biomarker studies should adjust for eGFR and albuminuria, while analyses of protein-bound toxins should also account for serum albumin. None of the candidate biomarkers currently has a universally accepted clinical reference range or a validated decision threshold.

**Table 3:** Summary of human interventional evidence relevant to the gut–kidney–NLRP3 axis.

Study or Intervention	Design and Population	Intervention and Duration	Principal Findings	Main Limitations
SYNERGY, NATUREN G, and ProLowCKD [83,84,85]	Small randomized trials in nondialysis CKD; 23–57 participants	Synbiotic- or probiotic-based interventions for 6 weeks to 3 months	SYNERGY reduced pCS but not IS. NATUREN G reduced free IS. ProLowCKD showed a favorable IS trend. No consistent eGFR effect was observed.	Small samples, short follow-up, different formulations, and predominantly surrogate endpoints.
SYNERGY II [86]	Double-blind feasibility RCT; 68 adults with CKD stages 3–4	Synbiotic versus placebo for 12 months	Microbial composition changed, but IS and pCS did not decrease. eGFR declined during follow-up.	Designed primarily for feasibility; not powered for kidney outcomes.
Mitrović et al., 2023 and 2024 [87,88]	Double-blind RCTs; 34 and 84 adults with eGFR 15–45 mL/min/1.73 m^2^	Multistrain synbiotic with inulin for 12 weeks	The 34-patient trial reduced IS and hsCRP and reported a short-term eGFR increase. The 84-patient conference report found reductions in IS, pCS, and hsCRP.	Single-center studies; short duration; the larger study is available only as a conference abstract.
Dehghani et al. [89]	Parallel RCT; 66 adults with CKD stages 3–4	Multistrain synbiotic with fructooligosaccharides for 6 weeks	Blood urea nitrogen decreased. Detailed toxin and eGFR results were not reported.	Short follow-up and limited extractable outcome data.
Planned multimodal study [90]	Prospective randomized study; planned enrollment of 200 participants with early CKD	Diet, exercise, and synbiotic intervention for 180 days	No results available.	NCT07483697.
AST-120 studies [91,92]	Randomized trial in 579 patients with advanced CKD; separate randomized open-label study in approximately 100 hemodialysis patients	Oral AST-120 for 36 months or 4 weeks	Long-term treatment did not improve renal or clinical outcomes. Short-term treatment reduced pruritus and transiently decreased IS, pCS, and TNF-α.	Discordant populations and endpoints; short-term biomarker changes do not establish disease modification.
Adsorptive PMMA hemodiafiltration [93]	Sequential feasibility study; 20 hemodialysis patients	PMMA-based hemodiafiltration for 6 months	IS and selected inflammatory markers decreased, whereas pCS did not.	Small, nonrandomized sequential design; no clinical outcome assessment.
AZD4144 clinical program [94,95,96]	Phase 1b randomized CKD/ASCVD study, *n* = 29; renal impairment pharmacokinetic study, *n* = 41	Oral selective NLRP3 inhibitor; single-dose or 28-day exposure	Studies were completed; publicly available efficacy results are not yet available.	Early-phase safety and pharmacokinetic studies; no long-term renal endpoints or concurrent toxin measurements.
				NCT06675175 and NCT06693765.

Abbreviations: ASCVD, atherosclerotic cardiovascular disease; CKD, chronic kidney disease; eGFR, estimated glomerular filtration rate; hsCRP, high-sensitivity C-reactive protein; IS, indoxyl sulfate; NLRP3, NOD-like receptor pyrin domain-containing protein 3; pCS, p-cresyl sulfate; PMMA, polymethylmethacrylate; RCT, randomized controlled trial; TNF-α, tumor necrosis factor alpha.

## Data Availability

No new data were created or analyzed in this study. Data sharing is not applicable to this article.

## Abbreviations

The following abbreviations are used in this manuscript:

16S rRNA	16S ribosomal RNA (ribonucleic acid)
AhR	aryl hydrocarbon receptor
AI	artificial intelligence
ASC	apoptosis-associated speck-like protein containing a caspase recruitment domain
ASCVD	atherosclerotic cardiovascular disease
ATP	adenosine triphosphate
CKD	chronic kidney disease
CRP	C-reactive protein
DNA	deoxyribonucleic acid
eGFR	estimated glomerular filtration rate
GSDMD	gasdermin D
HPLC	high-performance liquid chromatography
hsCRP	high-sensitivity C-reactive protein
IgA	immunoglobulin A
IKK	IκB kinase
IL	interleukin
IS	indoxyl sulfate
LC–MS/MS	liquid chromatography–tandem mass spectrometry
LPS	lipopolysaccharide
MAPK	mitogen-activated protein kinase
mTOR	mechanistic target of rapamycin
NADPH	nicotinamide adenine dinucleotide phosphate
NF-κB	nuclear factor kappa B
NLRP3	NOD-like receptor pyrin domain-containing protein 3
pCS	p-cresyl sulfate
PERK	protein kinase R-like endoplasmic reticulum kinase
PMMA	polymethylmethacrylate
RAAS	renin–angiotensin–aldosterone system
RCT	randomized controlled trial
ROS	reactive oxygen species
SCFA	short-chain fatty acid
SGLT2	sodium–glucose cotransporter 2
TGF-β	transforming growth factor beta
TLR4	Toll-like receptor 4
TMAO	trimethylamine N-oxide
TNF-α	tumor necrosis factor alpha
UACR	urinary albumin-to-creatinine ratio
ZO-1	zonula occludens-1

## References

[1] Mark P.B., Stafford L.K., Grams M.E., Aalruz H., Abd ElHafeez S., Abdelgalil A.A., Abdulkader R.S., Abeywickrama H.M., Abiodun O.O., Abramov D. (2025). Global, Regional, and National Burden of Chronic Kidney Disease in Adults, 1990–2023, and Its Attributable Risk Factors: A Systematic Analysis for the Global Burden of Disease Study 2023. Lancet.

[2] Gupta R., Woo K., Yi J.A. (2021). Epidemiology of End-Stage Kidney Disease. Semin. Vasc. Surg..

[3] Ravani P., Fiocco M., Liu P., Quinn R.R., Hemmelgarn B., James M., Lam N., Manns B., Oliver M.J., Strippoli G.F.M. (2019). Influence of Mortality on Estimating the Risk of Kidney Failure in People with Stage 4 CKD. J. Am. Soc. Nephrol..

[4] Barratt J. (2024). Mucosal Targeting in IgA Nephropathy Targeting the Gut Associated Lymphoid Tissue. Nephrology.

[5] Norouzi S., Lafayette R.A. (2026). Approved Therapies in the IgA Nephropathy Armamentarium: A Summary of the Evidence. Kidney Int. Suppl..

[6] Stamellou E., Bruchfeld A., Caravaca-Fontan F., Frangou E., Kronbichler A., Mirioglu S., Moran S.M., Stevens K.I., Teng Y.K.O., Steiger S. (2025). The 2025 KDIGO IgA Nephropathy Guideline Update: An ERA Immunonephrology Working Group Perspective. Clin. Kidney J..

[7] Lafayette R., Kristensen J., Stone A., Floege J., Tesař V., Trimarchi H., Zhang H., Eren N., Paliege A., Reich H.N. (2023). Efficacy and Safety of a Targeted-Release Formulation of Budesonide in Patients with Primary IgA Nephropathy (NefIgArd): 2-Year Results from a Randomised Phase 3 Trial. Lancet.

[8] Zhai Z., Huang Z., Wang L., Yu L., Gou R., Wang Y., Li Q., Guo Y., Tang L. (2026). Efficacy and Safety of Nefecon in IgA Nephropathy: Real World Clinical Practice. Front. Immunol..

[9] Rovin B.H., Barratt J., Cook H.T., Noronha I.L., Reich H.N., Suzuki Y., Tang S.C.W., Trimarch H., Floege J. (2025). KDIGO 2025 Clinical Practice Guideline for the Management of Immunoglobulin A Nephropathy (IgAN) and Immunoglobulin A Vasculitis (IgAV). Kidney Int..

[10] Rusu M., Ichim C., Anderco P., Pălăștea A., Boicean A. (2026). Gut-Kidney Axis: Unraveling the Role of the Microbiome in Chronic Kidney Disease. Biomedicines.

[11] Vilaysane A., Chun J., Seamone M.E., Wang W., Chin R., Hirota S., Li Y., Clark S.A., Tschopp J., Trpkov K. (2010). The NLRP3 Inflammasome Promotes Renal Inflammation and Contributes to CKD. J. Am. Soc. Nephrol..

[12] Komada T., Muruve D.A. (2019). The Role of Inflammasomes in Kidney Disease. Nat. Rev. Nephrol..

[13] Kim S.-M., Lee S.-H., Kim Y.-G., Kim S.-Y., Seo J.-W., Choi Y.-W., Kim D.-J., Jeong K.-H., Lee T.-W., Ihm C.-G. (2015). Hyperuricemia-Induced NLRP3 Activation of Macrophages Contributes to the Progression of Diabetic Nephropathy. Am. J. Physiol. Ren. Physiol..

[14] Ludwig-Portugall I., Bartok E., Dhana E., Evers B.D.G., Primiano M.J., Hall J.P., Franklin B.S., Knolle P.A., Hornung V., Hartmann G. (2016). An NLRP3-Specific Inflammasome Inhibitor Attenuates Crystal-Induced Kidney Fibrosis in Mice. Kidney Int..

[15] Yang X., Cai S., Gong J., Zhang J., Lian M., Chen R., Zhou L., Bai P., Liu B., Zhuang M. (2023). Characterization of Gut Microbiota in Patients with Stage 3–4 Chronic Kidney Disease: A Retrospective Cohort Study. Int. Urol. Nephrol..

[16] Vaziri N.D., Yuan J., Rahimi A., Ni Z., Said H., Subramanian V.S. (2012). Disintegration of Colonic Epithelial Tight Junction in Uremia: A Likely Cause of CKD-Associated Inflammation. Nephrol. Dial. Transplant..

[17] Zhao J., Ning X., Liu B., Dong R., Bai M., Sun S. (2021). Specific Alterations in Gut Microbiota in Patients with Chronic Kidney Disease: An Updated Systematic Review. Ren. Fail..

[18] Bauernfeind F.G., Horvath G., Stutz A., Alnemri E.S., MacDonald K., Speert D., Fernandes-Alnemri T., Wu J., Monks B.G., Fitzgerald K.A. (2009). Cutting Edge: NF-κB Activating Pattern Recognition and Cytokine Receptors License NLRP3 Inflammasome Activation by Regulating NLRP3 Expression. J. Immunol..

[19] Mihajlovic M., Krebber M.M., Yang Y., Ahmed S., Lozovanu V., Andreeva D., Verhaar M.C., Masereeuw R. (2021). Protein-Bound Uremic Toxins Induce Reactive Oxygen Species-Dependent and Inflammasome-Mediated IL-1β Production in Kidney Proximal Tubule Cells. Biomedicines.

[20] Wakamatsu T., Yamamoto S., Ito T., Sato Y., Matsuo K., Takahashi Y., Kaneko Y., Goto S., Kazama J., Gejyo F. (2018). Indoxyl Sulfate Promotes Macrophage IL-1β Production by Activating Aryl Hydrocarbon Receptor/NF-κ/MAPK Cascades, but the NLRP3 Inflammasome Was Not Activated. Toxins.

[21] Sun C.-Y., Hsu H.-H., Wu M.-S. (2013). P-Cresol Sulfate and Indoxyl Sulfate Induce Similar Cellular Inflammatory Gene Expressions in Cultured Proximal Renal Tubular Cells. Nephrol. Dial. Transplant..

[22] Chen Y.-Y., Chen D.-Q., Chen L., Liu J.-R., Vaziri N.D., Guo Y., Zhao Y.-Y. (2019). Microbiome–Metabolome Reveals the Contribution of Gut–Kidney Axis on Kidney Disease. J. Transl. Med..

[23] Młynarska E., Budny E., Saar M., Wojtanowska E., Jankowska J., Marciszuk S., Mazur M., Rysz J., Franczyk B. (2024). Does the Composition of Gut Microbiota Affect Chronic Kidney Disease? Molecular Mechanisms Contributed to Decreasing Glomerular Filtration Rate. Int. J. Mol. Sci..

[24] The Human Microbiome Project Consortium (2012). Structure, Function and Diversity of the Healthy Human Microbiome. Nature.

[25] Valdes A.M., Walter J., Segal E., Spector T.D. (2018). Role of the Gut Microbiota in Nutrition and Health. BMJ.

[26] Belkaid Y., Hand T.W. (2014). Role of the Microbiota in Immunity and Inflammation. Cell.

[27] Furusawa Y., Obata Y., Fukuda S., Endo T.A., Nakato G., Takahashi D., Nakanishi Y., Uetake C., Kato K., Kato T. (2013). Commensal Microbe-Derived Butyrate Induces the Differentiation of Colonic Regulatory T Cells. Nature.

[28] Koh A., De Vadder F., Kovatcheva-Datchary P., Bäckhed F. (2016). From Dietary Fiber to Host Physiology: Short-Chain Fatty Acids as Key Bacterial Metabolites. Cell.

[29] Parada Venegas D., De la Fuente M.K., Landskron G., González M.J., Quera R., Dijkstra G., Harmsen H.J.M., Faber K.N., Hermoso M.A. (2019). Short Chain Fatty Acids (SCFAs)-Mediated Gut Epithelial and Immune Regulation and Its Relevance for Inflammatory Bowel Diseases. Front. Immunol..

[30] Peng L., Li Z.-R., Green R.S., Holzmanr I.R., Lin J. (2009). Butyrate Enhances the Intestinal Barrier by Facilitating Tight Junction Assembly via Activation of AMP-Activated Protein Kinase in Caco-2 Cell Monolayers. J. Nutr..

[31] Turner J.R. (2009). Intestinal Mucosal Barrier Function in Health and Disease. Nat. Rev. Immunol..

[32] Chelakkot C., Ghim J., Ryu S.H. (2018). Mechanisms Regulating Intestinal Barrier Integrity and Its Pathological Implications. Exp. Mol. Med..

[33] Andersen K., Kesper M.S., Marschner J.A., Konrad L., Ryu M., Kumar Vr S., Kulkarni O.P., Mulay S.R., Romoli S., Demleitner J. (2017). Intestinal Dysbiosis, Barrier Dysfunction, and Bacterial Translocation Account for CKD–Related Systemic Inflammation. J. Am. Soc. Nephrol..

[34] Suliman I.L., Panculescu F.G., Fasie D., Cimpineanu B., Alexandru A., Gafar N., Popescu S., Nitu T.S., Enache F.-D., Chisnoiu T. (2025). Gut Microbiome in Patients with Chronic Kidney Disease Stages 4 and 5: A Systematic Literature Review. Int. J. Mol. Sci..

[35] Jiang S., Xie S., Lv D., Wang P., He H., Zhang T., Zhou Y., Lin Q., Zhou H., Jiang J. (2017). Alteration of the Gut Microbiota in Chinese Population with Chronic Kidney Disease. Sci. Rep..

[36] Souai N., Zidi O., Stathopoulou P., Bafoun A., Souiai O., Hanachi M., Asimakis E., Cherif A., Mosbah A., Tsiamis G. (2026). Gut Microbiota Signatures and Ecological Network Alterations Associated with Hemodialysis. Microorganisms.

[37] Mishima E., Fukuda S., Mukawa C., Yuri A., Kanemitsu Y., Matsumoto Y., Akiyama Y., Fukuda N.N., Tsukamoto H., Asaji K. (2017). Evaluation of the Impact of Gut Microbiota on Uremic Solute Accumulation by a CE-TOFMS–Based Metabolomics Approach. Kidney Int..

[38] Almeida P.P., Hoibian E., Stockler-Pinto M.B., Guebre-Egziabher F., Vidal H., Soulage C.O. (2026). Renal Failure-Driven Luminal Ammonia Production Impairs Gut Barrier Function in CKD. Biochimie.

[39] Guan R., Peng J., Gu Q., Xu J., Zhang J., Adamu H., Yang Q., Wang L., Cao G. (2026). The Gut-Kidney Axis in Chronic Kidney Disease: A Vicious Cycle of Microbial Dysbiosis and Uremic Toxin Accumulation. Front. Immunol..

[40] Knight R., Vrbanac A., Taylor B.C., Aksenov A., Callewaert C., Debelius J., Gonzalez A., Kosciolek T., McCall L.-I., McDonald D. (2018). Best Practices for Analysing Microbiomes. Nat. Rev. Microbiol..

[41] Lamba J.K., Tandon C., Tandon S. (2026). Gut Microbiota and Chronic Kidney Disease: A Complex Interplay with Implications for Diagnosis and Treatment. Probiotics Antimicrob. Proteins.

[42] Glorieux G., Gryp T., Perna A. (2020). Gut-Derived Metabolites and Their Role in Immune Dysfunction in Chronic Kidney Disease. Toxins.

[43] Abdel-Rahman S.M., Ozbek L., Narin A.E., Fidan D.G., Yilmaz Z.Y., Orak H.I., Guldan M., Kanbay M. (2026). Association between Interleukin-6, C-Reactive Protein and Chronic Kidney Disease Outcomes: A Systematic Review and Meta-Analysis. Eur. J. Intern. Med..

[44] Sánchez-Ospina D., Mas-Fontao S., Gracia-Iguacel C., Avello A., González De Rivera M., Mujika-Marticorena M., Gonzalez-Parra E. (2024). Displacing the Burden: A Review of Protein-Bound Uremic Toxin Clearance Strategies in Chronic Kidney Disease. J. Clin. Med..

[45] Iwasaki K., Miyoshi T., Urabe C., Sakuragi S., Kawai Y., Fuke S., Doi M., Takaishi A., Oka T., Tokunaga N. (2024). Impact of Serum Indoxyl Sulfate on One-Year Adverse Events in Chronic Kidney Disease Patients with Heart Failure. J. Clin. Med..

[46] Glorieux G., Vanholder R., Van Biesen W., Pletinck A., Schepers E., Neirynck N., Speeckaert M., De Bacquer D., Verbeke F. (2021). Free P-Cresyl Sulfate Shows the Highest Association with Cardiovascular Outcome in Chronic Kidney Disease. Nephrol. Dial. Transplant..

[47] Tang W.H.W., Wang Z., Kennedy D.J., Wu Y., Buffa J.A., Agatisa-Boyle B., Li X.S., Levison B.S., Hazen S.L. (2015). Gut Microbiota-Dependent Trimethylamine *N* -Oxide (TMAO) Pathway Contributes to Both Development of Renal Insufficiency and Mortality Risk in Chronic Kidney Disease. Circ. Res..

[48] Ahmed S., Sparidans R.W., Vernooij R.W.M., Mihaila S.M., Broekhuizen R., Goldschmeding R., Nguyen T.Q., Masereeuw R., Gerritsen K.G.F. (2025). Protein-Bound Uremic Toxin Clearance as Biomarker of Kidney Tubular Function in Diabetic Kidney Disease. Sci. Rep..

[49] Ribeiro A., Liu F., Srebrzynski M., Rother S., Adamowicz K., Wadowska M., Steiger S., Anders H.-J., Schmaderer C., Koziel J. (2023). Uremic Toxin Indoxyl Sulfate Promotes Macrophage-Associated Low-Grade Inflammation and Epithelial Cell Senescence. Int. J. Mol. Sci..

[50] Yamaguchi K., Yisireyili M., Goto S., Cheng X.W., Nakayama T., Matsushita T., Niwa T., Murohara T., Takeshita K. (2022). Indoxyl Sulfate Activates NLRP3 Inflammasome to Induce Cardiac Contractile Dysfunction Accompanied by Myocardial Fibrosis and Hypertrophy. Cardiovasc. Toxicol..

[51] Meijers B.K.I., De Loor H., Bammens B., Verbeke K., Vanrenterghem Y., Evenepoel P. (2009). P-Cresyl Sulfate and Indoxyl Sulfate in Hemodialysis Patients. Clin. J. Am. Soc. Nephrol..

[52] Huang W., Hung C., Yang C., Huang J. (2012). High Correlation Between Clearance of Renal Protein-Bound Uremic Toxins (Indoxyl Sulfate and *p*-Cresyl Sulfate) and Renal Water-Soluble Toxins in Peritoneal Dialysis Patients. Ther. Apher. Dial..

[53] Viaene L., Meijers B.K.I., Bammens B., Vanrenterghem Y., Evenepoel P. (2014). Serum Concentrations of p-Cresyl Sulfate and Indoxyl Sulfate, But Not Inflammatory Markers, Increase in Incident Peritoneal Dialysis Patients in Parallel with Loss of Residual Renal Function. Perit. Dial. Int. J. Int. Soc. Perit. Dial..

[54] Watanabe H., Miyamoto Y., Honda D., Tanaka H., Wu Q., Endo M., Noguchi T., Kadowaki D., Ishima Y., Kotani S. (2013). P-Cresyl Sulfate Causes Renal Tubular Cell Damage by Inducing Oxidative Stress by Activation of NADPH Oxidase. Kidney Int..

[55] Chern Y.-B., Chia K.L., Liu C.-H., Lin Y.-L., Tsai J.-P., Hsu B.-G. (2025). Serum P-Cresyl Sulfate Is Independently Associated with Aortic Stiffness in Non-Dialysis Chronic Kidney Disease Patients. Life.

[56] Hai X., Landeras V., Dobre M.A., DeOreo P., Meyer T.W., Hostetter T.H. (2015). Mechanism of Prominent Trimethylamine Oxide (TMAO) Accumulation in Hemodialysis Patients. PLoS ONE.

[57] Lai Y., Tang H., Zhang X., Zhou Z., Zhou M., Hu Z., Zhu F., Zhang L., Nie J. (2022). Trimethylamine-N-Oxide Aggravates Kidney Injury via Activation of P38/MAPK Signaling and Upregulation of HuR. Kidney Blood Press. Res..

[58] Kapetanaki S., Kumawat A.K., Persson K., Demirel I. (2021). The Fibrotic Effects of TMAO on Human Renal Fibroblasts Is Mediated by NLRP3, Caspase-1 and the PERK/Akt/mTOR Pathway. Int. J. Mol. Sci..

[59] Zhang X., Li Y., Yang P., Liu X., Lu L., Chen Y., Zhong X., Li Z., Liu H., Ou C. (2020). Trimethylamine-N-Oxide Promotes Vascular Calcification Through Activation of NLRP3 (Nucleotide-Binding Domain, Leucine-Rich-Containing Family, Pyrin Domain-Containing-3) Inflammasome and NF-κB (Nuclear Factor κB) Signals. Arterioscler. Thromb. Vasc. Biol..

[60] Fang L., Shen J., Xiao N., Tan Z. (2025). TMAO Activates the NLRP3 Inflammasome, Disrupts Gut–Kidney Interaction, and Promotes Intestinal Inflammation. Int. J. Mol. Sci..

[61] Chang D., Xu X., Yang Z., Ma T., Nie J., Dong J. (2022). Trimethylamine-N-Oxide (TMAO) and Clinical Outcomes in Patients with End-Stage Kidney Disease Receiving Peritoneal Dialysis. Perit. Dial. Int. J. Int. Soc. Perit. Dial..

[62] Lin C.-J., Wu V., Wu P.-C., Wu C.-J. (2015). Meta-Analysis of the Associations of p-Cresyl Sulfate (PCS) and Indoxyl Sulfate (IS) with Cardiovascular Events and All-Cause Mortality in Patients with Chronic Renal Failure. PLoS ONE.

[63] Broz P., Dixit V.M. (2016). Inflammasomes: Mechanism of Assembly, Regulation and Signalling. Nat. Rev. Immunol..

[64] Shi J., Gao W., Shao F. (2017). Pyroptosis: Gasdermin-Mediated Programmed Necrotic Cell Death. Trends Biochem. Sci..

[65] Mulay S.R., Kulkarni O.P., Rupanagudi K.V., Migliorini A., Darisipudi M.N., Vilaysane A., Muruve D., Shi Y., Munro F., Liapis H. (2013). Calcium Oxalate Crystals Induce Renal Inflammation by NLRP3-Mediated IL-1β Secretion. J. Clin. Investig..

[66] Morgan M.J., Liu Z. (2011). Crosstalk of Reactive Oxygen Species and NF-κB Signaling. Cell Res..

[67] Ho L.-C., Wu T.-Y., Lin T.-M., Liou H.-H., Hung S.-Y. (2021). Indoxyl Sulfate Mediates the Low Inducibility of the NLRP3 Inflammasome in Hemodialysis Patients. Toxins.

[68] Chen M.-L., Zhu X.-H., Ran L., Lang H.-D., Yi L., Mi M.-T. (2017). Trimethylamine-N-Oxide Induces Vascular Inflammation by Activating the NLRP3 Inflammasome Through the SIRT3-SOD2-mtROS Signaling Pathway. J. Am. Heart Assoc..

[69] Kishi S., Nagasu H., Kidokoro K., Kashihara N. (2024). Oxidative Stress and the Role of Redox Signalling in Chronic Kidney Disease. Nat. Rev. Nephrol..

[70] Frąk W., Dąbek B., Balcerczyk-Lis M., Motor J., Radzioch E., Młynarska E., Rysz J., Franczyk B. (2024). Role of Uremic Toxins, Oxidative Stress, and Renal Fibrosis in Chronic Kidney Disease. Antioxidants.

[71] More S.A., Patil A., Joshi M., Dhokne P., Nimbarte V.D., Mulay S.R. (2026). The NLRP3 Inflammasome in Chronic Kidney Disease Progression: Systematic Review of Preclinical Models Reveals a Critical Gap in Pyroptosis Research and Therapeutic Translation. Mol. Biol. Rep..

[72] Harlacher E., Wollenhaupt J., Baaten C.C.F.M.J., Noels H. (2022). Impact of Uremic Toxins on Endothelial Dysfunction in Chronic Kidney Disease: A Systematic Review. Int. J. Mol. Sci..

[73] Jia M., Lin L., Xun K., Li D., Wu W., Sun S., Qiu H., Jin D. (2024). Indoxyl Sulfate Aggravates Podocyte Damage through the TGF-Β1/Smad/ROS Signaling Pathway. Kidney Blood Press. Res..

[74] Kuppe C., Ibrahim M.M., Kranz J., Zhang X., Ziegler S., Perales-Patón J., Jansen J., Reimer K.C., Smith J.R., Dobie R. (2021). Decoding Myofibroblast Origins in Human Kidney Fibrosis. Nature.

[75] Wharram B.L., Goyal M., Wiggins J.E., Sanden S.K., Hussain S., Filipiak W.E., Saunders T.L., Dysko R.C., Kohno K., Holzman L.B. (2005). Podocyte Depletion Causes Glomerulosclerosis: Diphtheria Toxin–Induced Podocyte Depletion in Rats Expressing Human Diphtheria Toxin Receptor Transgene. J. Am. Soc. Nephrol..

[76] Mirzayi C., Renson A., Furlanello C., Sansone S.-A., Zohra F., Elsafoury S., Geistlinger L., Kasselman L.J., Genomic Standards Consortium, Massive Analysis and Quality Control Society (2021). Reporting Guidelines for Human Microbiome Research: The STORMS Checklist. Nat. Med..

[77] Al-Dajani A.R., Hou Q.K., Kiang T.K.L. (2024). Liquid Chromatography-Mass Spectrometry Analytical Methods for the Quantitation of p-Cresol Sulfate and Indoxyl Sulfate in Human Matrices: Biological Applications and Diagnostic Potentials. Pharmaceutics.

[78] Lei Y., Jiang J., Chen L., Fang Q., Li C., Fei Y., Qu S., Xia Z., Ning Y., He M. (2025). Utility of Trimethylamine N-Oxide (TMAO) as a Biomarker for Risk Stratification in Chronic Kidney Disease: A Multicenter Observational Study. BMC Nephrol..

[79] Nagar A., Bharadwaj R., Shaikh M.O.F., Roy A. (2023). What Are NLRP3-ASC Specks? An Experimental Progress of 22 Years of Inflammasome Research. Front. Immunol..

[80] Chern Y.-B., Tsai J.-P., Liu C.-H., Lin Y.-L., Wang C.-H., Hsu B.-G. (2025). Serum Indoxyl Sulfate as a Potential Biomarker of Peripheral Arterial Stiffness in Patients with Non-Dialysis Chronic Kidney Disease Stages 3 to 5. Toxins.

[81] Chen Z., Xu J., Xing X., Xue C., Luo X., Gao S., Mao Z. (2022). P-Cresyl Sulfate Predicts Clinical Outcomes in Sustained Peritoneal Dialysis: A 5-Year Follow-up Cohort Study and Meta-Analysis. Ren. Fail..

[82] Pelletier C.C., Croyal M., Ene L., Aguesse A., Billon-Crossouard S., Krempf M., Lemoine S., Guebre-Egziabher F., Juillard L., Soulage C.O. (2019). Elevation of Trimethylamine-N-Oxide in Chronic Kidney Disease: Contribution of Decreased Glomerular Filtration Rate. Toxins.

[83] Rossi M., Johnson D.W., Morrison M., Pascoe E.M., Coombes J.S., Forbes J.M., Szeto C.-C., McWhinney B.C., Ungerer J.P.J., Campbell K.L. (2016). Synbiotics Easing Renal Failure by Improving Gut Microbiology (SYNERGY): A Randomized Trial. Clin. J. Am. Soc. Nephrol..

[84] Cosola C., Rocchetti M.T., Di Bari I., Acquaviva P.M., Maranzano V., Corciulo S., Di Ciaula A., Di Palo D.M., La Forgia F.M., Fontana S. (2021). An Innovative Synbiotic Formulation Decreases Free Serum Indoxyl Sulfate, Small Intestine Permeability and Ameliorates Gastrointestinal Symptoms in a Randomized Pilot Trial in Stage IIIb-IV CKD Patients. Toxins.

[85] De Mauri A., Carrera D., Bagnati M., Rolla R., Vidali M., Chiarinotti D., Pane M., Amoruso A., Del Piano M. (2022). Probiotics-Supplemented Low-Protein Diet for Microbiota Modulation in Patients with Advanced Chronic Kidney Disease (ProLowCKD): Results from a Placebo-Controlled Randomized Trial. Nutrients.

[86] McFarlane C., Krishnasamy R., Stanton T., Savill E., Snelson M., Mihala G., Kelly J.T., Morrison M., Johnson D.W., Campbell K.L. (2021). Synbiotics Easing Renal Failure by Improving Gut Microbiology II (SYNERGY II): A Feasibility Randomized Controlled Trial. Nutrients.

[87] Mitrović M., Stanković-Popović V., Tolinački M., Golić N., Soković Bajić S., Veljović K., Nastasijević B., Soldatović I., Svorcan P., Dimković N. (2023). The Impact of Synbiotic Treatment on the Levels of Gut-Derived Uremic Toxins, Inflammation, and Gut Microbiome of Chronic Kidney Disease Patients—A Randomized Trial. J. Ren. Nutr..

[88] Mitrović M., Dimkovic N., Popovic V.S. (2024). #279 The Impact of Synbiotics on the Uremic Toxins, Inflammation, and Gut Microbiome of CKD Patients—Randomized, Double-Blind, Placebo-Controlled Trial. Nephrol. Dial. Transplant..

[89] Dehghani H., Heidari F., Mozaffari-Khosravi H., Nouri-Majelan N., Dehghani A. (2016). Synbiotic Supplementations for Azotemia in Patients with Chronic Kidney Disease: A Randomized Controlled Trial. Iran. J. Kidney Dis..

[90] Hernandez K.V.P. (2026). Multimodal Intervention and Its Association with Reduced Dysbiosis and Inflammation in Early Chronic Kidney Disease in Young People: A Prospective Study.

[91] Cha R.-H., Kang S.W., Park C.W., Cha D.R., Na K.Y., Kim S.G., Yoon S.A., Han S.Y., Chang J.H., Park S.K. (2016). A Randomized, Controlled Trial of Oral Intestinal Sorbent AST-120 on Renal Function Deterioration in Patients with Advanced Renal Dysfunction. Clin. J. Am. Soc. Nephrol..

[92] Wu C.-C., Tian Y.-C., Lu C.-L., Wu M.-J., Lim P.-S., Chiu Y.-W., Kuo K.-L., Liu S.-H., Chou Y.-C., Sun C.-A. (2024). AST-120 Improved Uremic Pruritus by Lowering Indoxyl Sulfate and Inflammatory Cytokines in Hemodialysis Patients. Aging.

[93] Fabbrini P., Vergani D., Malinverno A., Pieruzzi F., Marengo M., Merlotti G., Medana C., Quercia A.D., Cantaluppi V. (2023). Protein-Bound Uremic Toxins and Inflammation Process in Hemodialysis Patients: Is There a Role for Adsorption Hemodiafiltration?. Blood Purif..

[94] Olson S.W., Pearce A., Schaffner H., Varma V., Egeland E.J., Eketjäll S., MacPhee I. (2025). Safety, Tolerability, Pharmacokinetics, and Target Engagement of Single and Multiple Ascending Doses of Intravenous AZD4144, a Small Molecule NLRP3 Inhibitor for Treatment of AKI. J. Am. Soc. Nephrol..

[95] AstraZeneca (2025). A Phase I, Randomized, Double-Blind, Placebo-Controlled, Multi-Centre Study to Assess the Safety, Tolerability, and Pharmacodynamics of AZD4144 in Participants with Established Atherosclerotic Cardiovascular and Chronic Kidney Disease.

[96] AstraZeneca (2026). A Single Dose, Non-Randomised, Open-Label, Parallel Group Study to Assess the Pharmacokinetics, Safety, and Tolerability of AZD4144 in Participants with Severe Renal Impairment, End-Stage Kidney Disease, and in Healthy Participants.

[97] Nguyen T.T.U., Kim H.W., Kim W. (2021). Effects of Probiotics, Prebiotics, and Synbiotics on Uremic Toxins, Inflammation, and Oxidative Stress in Hemodialysis Patients: A Systematic Review and Meta-Analysis of Randomized Controlled Trials. J. Clin. Med..

[98] Cedillo-Flores R., Cuevas-Budhart M.A., Cavero-Redondo I., Kappes M., Ávila-Díaz M., Paniagua R. (2025). Impact of Gut Microbiome Modulation on Uremic Toxin Reduction in Chronic Kidney Disease: A Systematic Review and Network Meta-Analysis. Nutrients.

[99] Hsu C.-K., Chang L.-C., Chen Y.-T., Chen C.-Y., Hsu H.-R., Bai S., Lee C.-C., Jangir H., Sun C.-Y., Su S.-C. (2025). Effects of Sodium-Glucose Cotransporter-2 Inhibitors on Modulating Protein-Bound Uremic Toxins and Gut Microbiota in Predialysis CKD Patients: Matched Case-Control Study. Kidney360.

[100] Van Bommel E.J.M., Herrema H., Davids M., Kramer M.H.H., Nieuwdorp M., Van Raalte D.H. (2020). Effects of 12-Week Treatment with Dapagliflozin and Gliclazide on Faecal Microbiome: Results of a Double-Blind Randomized Trial in Patients with Type 2 Diabetes. Diabetes Metab..

[101] Liu C., Yang L., Wei W., Fu P. (2024). Efficacy of Probiotics/Synbiotics Supplementation in Patients with Chronic Kidney Disease: A Systematic Review and Meta-Analysis of Randomized Controlled Trials. Front. Nutr..

[102] Li Y.-K., Li W.-R., Ren H., Xiao C.-L., Guo Z., Luo J.-Q. (2025). Gut Microbiome-Targeted Therapeutics for Chronic Kidney Disease: Comparative Efficacy of Probiotic and Microbial Preparations. Inflammopharmacology.

[103] Wathanavasin W., Jaturapisanukul S., Janwetchasil P., Thongprayoon C., Cheungpasitporn W., Fülöp T. (2026). Effects of Hemodiafiltration Versus Hemodialysis on Uremic Toxins, Inflammatory Markers, Anemia, and Nutritional Parameters: A Systematic Review and Meta-Analysis. Toxins.

[104] Takkavatakarn K., Wuttiputinun T., Phannajit J., Praditpornsilpa K., Eiam-Ong S., Susantitaphong P. (2021). Protein-Bound Uremic Toxin Lowering Strategies in Chronic Kidney Disease: A Systematic Review and Meta-Analysis. J. Nephrol..

[105] Johansson A., Sugama H., Larsson N., Winiwarter S., Yoshida K., Gradén H., Bergonzini G., Matsumura T., Miyazaki S., Fuchigami R. (2025). Discovery of AZD4144, a Selective and Potent NLRP3 Inhibitor for the Treatment of Inflammatory Diseases. J. Med. Chem..

[106] Da Cunha R.S., Gregório P.C., Morais A.C.C., Barreto F.C., Stinghen A.E.M. (2026). Targeting Uremic Toxins in Chronic Kidney Disease: Current Challenges and Emerging Therapeutic Strategies. Toxins.

[107] Barreto F.C., Barreto D.V., Liabeuf S., Meert N., Glorieux G., Temmar M., Choukroun G., Vanholder R., Massy Z.A. (2009). Serum Indoxyl Sulfate Is Associated with Vascular Disease and Mortality in Chronic Kidney Disease Patients. Clin. J. Am. Soc. Nephrol..

[108] Liabeuf S., Barreto D.V., Barreto F.C., Meert N., Glorieux G., Schepers E., Temmar M., Choukroun G., Vanholder R., Massy Z.A. (2010). Free P-Cresylsulphate Is a Predictor of Mortality in Patients at Different Stages of Chronic Kidney Disease. Nephrol. Dial. Transplant..

[109] Missailidis C., Hällqvist J., Qureshi A.R., Barany P., Heimbürger O., Lindholm B., Stenvinkel P., Bergman P. (2016). Serum Trimethylamine-N-Oxide Is Strongly Related to Renal Function and Predicts Outcome in Chronic Kidney Disease. PLoS ONE.

[110] Vanholder R., Schepers E., Pletinck A., Nagler E.V., Glorieux G. (2014). The Uremic Toxicity of Indoxyl Sulfate and P-Cresyl Sulfate: A Systematic Review. J. Am. Soc. Nephrol..

[111] Ramezani A., Massy Z.A., Meijers B., Evenepoel P., Vanholder R., Raj D.S. (2016). Role of the Gut Microbiome in Uremia: A Potential Therapeutic Target. Am. J. Kidney Dis..

[112] Meijers B.K.I., Evenepoel P. (2011). The Gut-Kidney Axis: Indoxyl Sulfate, p-Cresyl Sulfate and CKD Progression. Nephrol. Dial. Transplant..

[113] Anders H.-J., Andersen K., Stecher B. (2013). The Intestinal Microbiota, a Leaky Gut, and Abnormal Immunity in Kidney Disease. Kidney Int..

[114] Castillo-Rodriguez E., Fernandez-Prado R., Esteras R., Perez-Gomez M.V., Gracia-Iguacel C., Fernandez-Fernandez B., Kanbay M., Tejedor A., Lazaro A., Ruiz-Ortega M. (2018). Impact of Altered Intestinal Microbiota on Chronic Kidney Disease Progression. Toxins.

[115] Schroder K., Tschopp J. (2010). The Inflammasomes. Cell.

[116] Tschopp J., Schroder K. (2010). NLRP3 Inflammasome Activation: The Convergence of Multiple Signalling Pathways on ROS Production?. Nat. Rev. Immunol..

[117] Kelley N., Jeltema D., Duan Y., He Y. (2019). The NLRP3 Inflammasome: An Overview of Mechanisms of Activation and Regulation. Int. J. Mol. Sci..

[118] Jo E.-K., Kim J.K., Shin D.-M., Sasakawa C. (2016). Molecular Mechanisms Regulating NLRP3 Inflammasome Activation. Cell. Mol. Immunol..

[119] Anders H.-J., Muruve D.A. (2011). The Inflammasomes in Kidney Disease. J. Am. Soc. Nephrol..

[120] Dinarello C.A. (2018). Overview of the IL-1 Family in Innate Inflammation and Acquired Immunity. Immunol. Rev..

[121] Proctor L.M., Creasy H.H., Fettweis J.M., Lloyd-Price J., Mahurkar A., Zhou W., Buck G.A., Snyder M.P., Strauss J.F., The Integrative HMP (iHMP) Research Network Consortium (2019). The Integrative Human Microbiome Project. Nature.

[122] Gilbert J.A., Blaser M.J., Caporaso J.G., Jansson J.K., Lynch S.V., Knight R. (2018). Current Understanding of the Human Microbiome. Nat. Med..

[123] Guo H., Callaway J.B., Ting J.P.-Y. (2015). Inflammasomes: Mechanism of Action, Role in Disease, and Therapeutics. Nat. Med..

[124] Li Y., Lu H., Guo J., Zhang M., Zheng H., Liu Y., Liu W. (2023). Gut Microbiota-Derived Trimethylamine N-Oxide Is Associated with the Risk of All-Cause and Cardiovascular Mortality in Patients with Chronic Kidney Disease: A Systematic Review and Dose-Response Meta-Analysis. Ann. Med..

[125] Meijers B.K.I., Claes K., Bammens B., De Loor H., Viaene L., Verbeke K., Kuypers D., Vanrenterghem Y., Evenepoel P. (2010). P-Cresol and Cardiovascular Risk in Mild-to-Moderate Kidney Disease. Clin. J. Am. Soc. Nephrol..

[126] Poesen R., Viaene L., Verbeke K., Augustijns P., Bammens B., Claes K., Kuypers D., Evenepoel P., Meijers B. (2014). Cardiovascular Disease Relates to Intestinal Uptake of P-Cresol in Patients with Chronic Kidney Disease. BMC Nephrol..

[127] Janeiro M., Ramírez M., Milagro F., Martínez J., Solas M. (2018). Implication of Trimethylamine N-Oxide (TMAO) in Disease: Potential Biomarker or New Therapeutic Target. Nutrients.

[128] Kanbay M., Al-Shiab R., Shah E., Ozbek L., Guldan M., Ortiz A., Fouque D. (2025). Gut Microbiota Modulation in GLP-1RA and SGLT-2i Therapy: Clinical Implications and Mechanistic Insights in Type 2 Diabetes. Clin. Kidney J..

[129] Tao Y., Zhang N., Wang Z., Pan Y., Zhong S., Liu H. (2025). SGLT2 Inhibitors Confer Cardiovascular Protection via the Gut-Kidney-Heart Axis: Mechanisms and Translational Perspectives. J. Cardiovasc. Dev. Dis..

[130] George M.Y., Gamal N.K., Safwat K., Mamdouh M., AbdElFatah A., Atallah A., Cerchione C. (2026). From Glucose Transport to Microbial Modulation: The Impact of Sodium Glucose Co-Transporter-2 Inhibitors on the Gut Microbiota. Med. Sci..

[131] Sirich T.L., Plummer N.S., Gardner C.D., Hostetter T.H., Meyer T.W. (2014). Effect of Increasing Dietary Fiber on Plasma Levels of Colon-Derived Solutes in Hemodialysis Patients. Clin. J. Am. Soc. Nephrol..

[132] Marzocco S., Dal Piaz F., Di Micco L., Torraca S., Sirico M.L., Tartaglia D., Autore G., Di Iorio B. (2013). Very Low Protein Diet Reduces Indoxyl Sulfate Levels in Chronic Kidney Disease. Blood Purif..

[133] Borges N.A., Carmo F.L., Stockler-Pinto M.B., De Brito J.S., Dolenga C.J., Ferreira D.C., Nakao L.S., Rosado A., Fouque D., Mafra D. (2018). Probiotic Supplementation in Chronic Kidney Disease: A Double-Blind, Randomized, Placebo-Controlled Trial. J. Ren. Nutr..

[134] Noel S., Martina-Lingua M.N., Bandapalle S., Pluznick J., Hamad A.R.A., Peterson D.A., Rabb H. (2014). Intestinal Microbiota-Kidney Cross Talk in Acute Kidney Injury and Chronic Kidney Disease. Nephron Clin. Pract..

[135] McFarlane C., Ramos C.I., Johnson D.W., Campbell K.L. (2019). Prebiotic, Probiotic, and Synbiotic Supplementation in Chronic Kidney Disease: A Systematic Review and Meta-Analysis. J. Ren. Nutr..

[136] Lowenstein J., Nigam S.K. (2021). Uremic Toxins in Organ Crosstalk. Front. Med..

[137] Lin C.-J., Liou T.-C., Pan C.-F., Wu P.-C., Sun F.-J., Liu H.-L., Chen H.-H., Wu C.-J. (2015). The Role of Liver in Determining Serum Colon-Derived Uremic Solutes. PLoS ONE.

[138] Su J.-Q., Wu X.-Q., Wang Q., Xie B.-Y., Xiao C.-Y., Su H.-Y., Tang J.-X., Yao C.-W. (2025). The Microbial Metabolite Trimethylamine N-Oxide and the Kidney Diseases. Front. Cell. Infect. Microbiol..

[139] Bogdan R.G., Boicean A., Anderco P., Ichim C., Iliescu-Glaja M., Todor S.B., Leonte E., Bloanca V.A., Crainiceanu Z.P., Popa M.L. (2025). From Liver to Kidney: The Overlooked Burden of Nonalcoholic Fatty Liver Disease in Chronic Kidney Disease. J. Clin. Med..

[140] Lekawanvijit S., Kompa A.R., Wang B.H., Kelly D.J., Krum H. (2012). Cardiorenal Syndrome: The Emerging Role of Protein-Bound Uremic Toxins. Circ. Res..

[141] Rysz J., Franczyk B., Ławiński J., Olszewski R., Ciałkowska-Rysz A., Gluba-Brzózka A. (2021). The Impact of CKD on Uremic Toxins and Gut Microbiota. Toxins.

[142] Riley R.D., Archer L., Snell K.I.E., Ensor J., Dhiman P., Martin G.P., Bonnett L.J., Collins G.S. (2024). Evaluation of Clinical Prediction Models (Part 2): How to Undertake an External Validation Study. BMJ.

[143] Borta S.M., Crișan A.S., Popețiu R.O., Vulciu P.A., Știrbu O., Avram C.R., Goldiș D., Rus L.A., Roman D.R., Chioreanu A. (2026). From Airway Inflammation to Molecular Remodeling: Integrating YKL-40, MBL and Epigenetic Biomarkers in Asthma and COPD. Biomolecules.

[144] Donath-Miklos I., Popețiu R.O., Crișan A.S., Vulciu P.A., Știrbu O., Coman R.A., Avram C., Goldiș D., Roman D.R., Chioreanu A. (2026). Inflammatory and Epigenetic Biomarkers in Prostate Disease: Current Evidence, Clinical Limitations, and Future Multimarker Strategies. Diagnostics.

